# Aging is associated with increased chromatin accessibility and reduced polymerase pausing in liver

**DOI:** 10.15252/msb.202211002

**Published:** 2022-09-09

**Authors:** Mihaela Bozukova, Chrysa Nikopoulou, Niklas Kleinenkuhnen, Dora Grbavac, Katrin Goetsch, Peter Tessarz

**Affiliations:** ^1^ Max Planck Research Group ‘Chromatin and Ageing’ Max Planck Institute for Biology of Ageing Cologne Germany; ^2^ Faculty of Medicine, Institute of Medical Statistics and Computational Biology University of Cologne Cologne Germany; ^3^ Cellular Stress Responses in Aging‐Associated Diseases (CECAD) University of Cologne Cologne Germany; ^4^ Present address: Center for Integrative Genomics University of Lausanne Lausanne Switzerland

**Keywords:** aging, chromatin architecture, nascent transcription, promoter‐proximal pausing, Chromatin, Transcription & Genomics

## Abstract

Regulation of gene expression is linked to the organization of the genome. With age, chromatin alterations occur on all levels of genome organization, accompanied by changes in the gene expression profile. However, little is known about the changes in the level of transcriptional regulation. Here, we used a multi‐omics approach and integrated ATAC‐, RNA‐ and NET‐seq to identify age‐related changes in the chromatin landscape of murine liver and to investigate how these are linked to transcriptional regulation. We provide the first systematic inventory of the connection between aging, chromatin accessibility, and transcriptional regulation in a whole tissue. Aging in murine liver is characterized by an increase in chromatin accessibility at promoter regions, but not in an increase in transcriptional output. Instead, aging is accompanied by a decrease in promoter‐proximal pausing of RNA polymerase II (Pol II), while initiation of transcription is not decreased as assessed by RNA polymerase mapping using CUT&RUN. Based on the data reported, we propose that these age‐related changes in transcriptional regulation are due to a reduced stability of the pausing complex.

## Graphical abstract

Profiling the chromatin landscape and nascent transcriptome in murine liver reveals an age‐related increase in chromatin accessibility at promoters and enhancers and a decrease in promoter‐proximal pausing of RNA polymerase II due to decreased pausing complex stability.

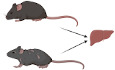

## Introduction

Epigenetic drifts as well as changes in chromatin architecture and transcriptional programs commonly occur during mammalian aging (Benayoun *et al*, 2015, 2019; Maleszewska *et al*, 2016) and contribute to the general decline in physiological function that is usually observed in older individuals (López‐Otín *et al*, 2013). The importance of epigenetic and transcriptional alterations in the aging process is underscored by several recent studies that employed partial reprogramming—an inherently epigenetic phenomenon—to rejuvenate several tissues in a variety of different aging models. Such rejuvenation phenomena do not only positively impact physiology, but also established a more youthful epigenetic state as measured by changes in the DNA methylation pattern (Lu *et al*, 2020; preprint: Chondronasiou *et al*, 2022), the levels of histone modifications (Ocampo *et al*, 2016), and the transcriptome itself (Lu *et al*, 2020). In summary, epigenetic and transcriptional states change with age and are directly linked to physiological fitness. Importantly, however, all studies addressing aging conducted up to now have investigated transcriptional changes using either microarrays or RNA‐seq. These technologies measure steady‐state levels of mRNA, which depend on the rate of nascent transcription and mRNA degradation (Nikopoulou *et al*, 2019)—both of which might change upon aging. Recent advances have made it possible to directly monitor nascent transcripts, using approaches such as GRO‐seq (Core *et al*, 2008) and NET‐seq (Mayer *et al*, 2015), but so far, these technologies have been only used in tissue culture.

Local chromatin architecture is an important regulator of transcription (Venkatesh & Workman, 2015). Nucleosomes create physical barriers to RNA polymerase (Pol II) and transcription factors (Petesch & Lis, 2012). Thus, for initiation of transcription, the promoter region needs to be rendered accessible, which is achieved by the combined action of pioneering transcription factors (TFs) (Zaret & Carroll, 2011) and chromatin remodelers (Venkatesh & Workman, 2015). The size of the nucleosome‐free region positively correlates with TF binding and subsequent gene activation (Scruggs *et al*, 2015). During transcription initiation, Pol II is recruited to the accessible promoter region through the concerted action of general transcription factors, resulting in the sequential formation of the preinitiation complex (PIC), before phosphorylation of Ser5 and Ser7 of the C‐terminal domain (CTD) of Pol II triggers promoter escape (Wong *et al*, 2014). However, Pol II stalls after the initial transcription of about 20–60 nucleotides, in a process termed promoter‐proximal pausing. Over the last decade, genome‐wide studies have demonstrated that Pol II pausing is a ubiquitous step in the transcription cycle of Drosophila and mammalian genes (Muse *et al*, 2007; Zeitlinger *et al*, 2007), which serves as an additional regulatory layer. Paused polymerase is thought to allow further fine‐tuning of transcription and the integration of various upstream signals. Indeed, ES cells that have decreased pausing efficiency became refractory to differentiation cues, highlighting the role of pausing in regulating signaling cascades in response to differentiation cues (Williams *et al*, 2015). In addition, paused Pol II is important to maintain nucleosome‐depleted regions around promoters (Core & Adelman, 2019). Stable promoter‐proximal Pol II pausing is facilitated by binding of the pause‐inducing factors DSIF (DRB sensitivity‐inducing factor), a heterodimer consisting of SPT4 and SPT5 as well as NELF (negative elongation factor) to Pol II (Wada *et al*, 1998; Wu *et al*, 2003; Lee *et al*, 2008). To release paused Pol II into productive elongation, CDK9 as part of the of P‐TEFb complex (Marshall & Price, 1995), phosphorylates NELF, the DSIF subunit SPT5 as well as Ser2 of the CTD of Pol II. SPT5 phosphorylation transforms DSIF from an inhibitory to a stimulating elongation factor (Bernecky *et al*, 2017) and triggers the dissociation of NELF and progression of Pol II into productive elongation.

Here, we analyzed how local chromatin structure and transcription initiation are altered in the aging liver using ATAC‐seq together with a NET‐seq protocol adapted for tissues and in combination with publicly available RNA‐ and ChIP‐seq data. We demonstrate that aging in the liver is characterized by a strong increase in promoter accessibility. However, this increase in accessibility is not reflected in an increased transcriptional output on nascent or steady‐state level. Interestingly, the overlap of differentially regulated steady‐state and nascent RNA is minor, indicating that post‐transcriptional processes play a major role in shaping the transcriptome during the aging process. Despite the overall increase in promoter accessibility, we show that the levels of promoter‐proximal pausing globally decline with age. However, this is not due to reduced polymerase loading as assessed by CUT&RUN. On the other hand, mapping of SPT4 binding to chromatin revealed that DSIF is recruited less efficiently to the promoter‐proximal region in aged livers. Furthermore, several lines of evidence suggest that transcriptional initiation is unaltered with age. Together, these data indicate a general loss of stability of the Pol II pausing complex upon aging, which we propose might mitigate the effects of the overall increase in promoter accessibility to maintain a relatively young‐like transcription state in the aging liver.

## Results

### Promoter regions are more accessible with age

**Figure 1:**
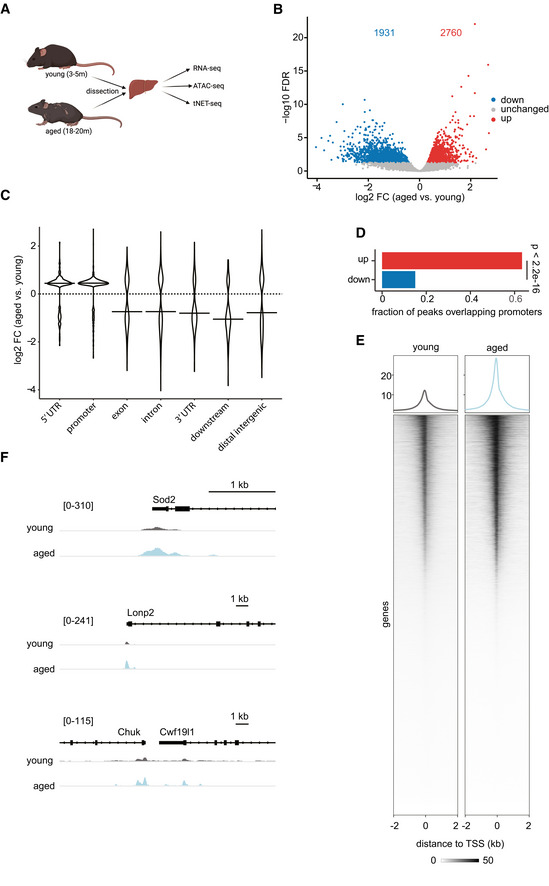
Chromatin accessibility at promoter regions increases with age in murine liver tissue ASchematic of experimental setup.BVolcano plot of differentially accessible genomic regions comparing liver tissue of aged relative to young mice (FDR < 0.05, Wald test). Increased accessibility: 2,760 regions (red), decreased accessibility: 1,931 regions (blue), regions with unchanged accessibility: 50,978 (gray).CGenomic locations of differentially accessible sites. *Y*‐axis represents the log2‐fold change (log2 FC) in accessibility in the liver of aged versus young animals. Horizontal bar indicates median of the data.DProportion of differentially accessible sites overlapping promoters (defined as TSS ±200 bp). *P*‐value was calculated using a two‐sided, two‐proportion *z*‐test.EHeatmap and average intensity profiles of promoter accessibility over all annotated TSSs in the mouse genome. Read densities of merged biological replicates (*n* = 3 young and 4 aged) were normalized to 1× coverage.FRepresentative genome browser views of promoters with increased accessibility in aged mice. Peak intensity range is indicated in brackets. Reads from biological replicates were merged and normalized to 1× coverage.

**Figure EV1:**
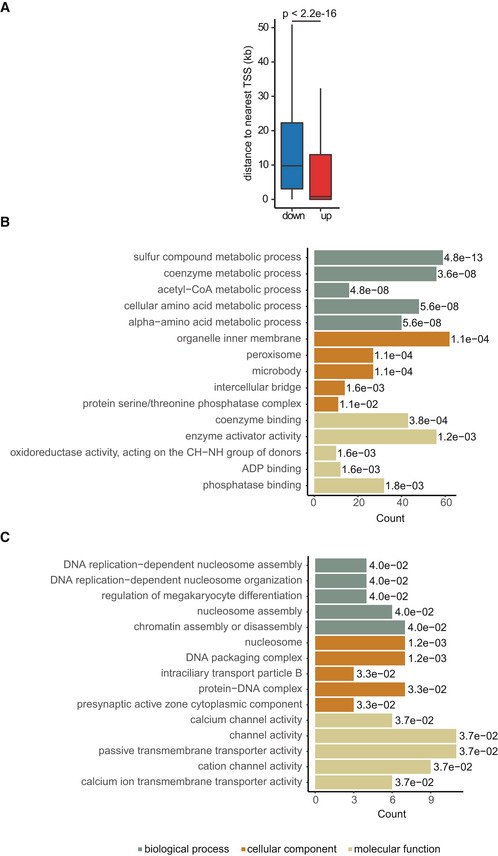
Promoter accessibility and functional annotation ADistance of differentially accessible sites to the nearest annotated TSS. *P*‐value was calculated using a two‐sided Wilcoxon rank‐sum test. *N* = 2,760 and 1,931 sites with increased and decreased accessibility with age, respectively. Box plots consist of the median (central line), the 25^th^ and 75^th^ percentiles (box) and the highest/lowest value within 1.5 * interquartile range of the box (whiskers).B, CGO term enrichment analysis for genes with increased (B) and decreased (C) promoter accessibility in the liver of aged mice. Only genes with a differentially accessible TSS were included in the analysis (*n* = 1,945 genes in total; 1,704 with increased and 241 with decreased promoter accessibility). Only the top five enriched GO terms ranked by adjusted *P*‐value (Benjamini‐Hochberg [BH] procedure, FDR < 0.05) are displayed. BH‐adjusted *P*‐values are reported for each GO term inside the respective bar. Data information: ATAC‐seq: young, *n* = 3; old, *n* = 4; all biological replicates.

**Figure EV2:**
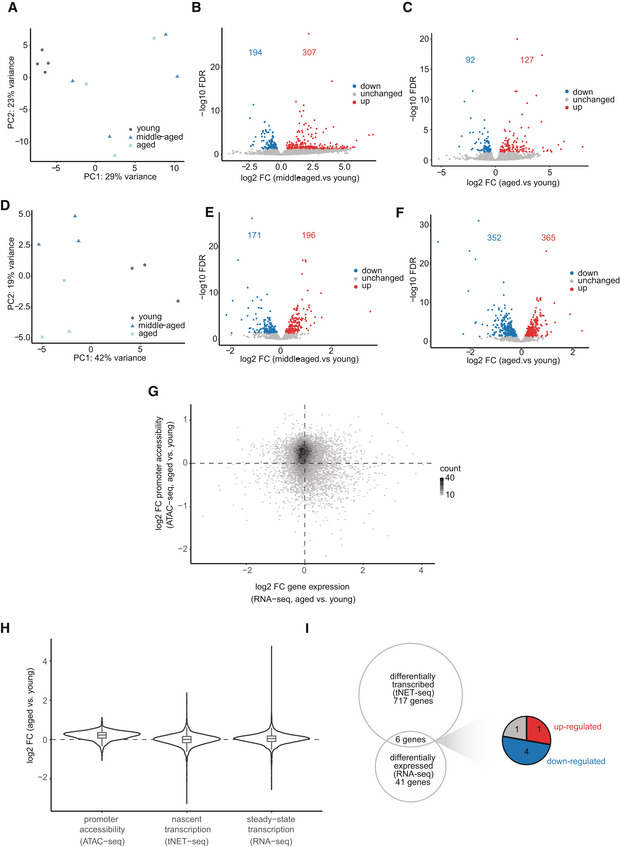
Integration of RNA‐ and (t)NET‐seq APCA scatter plot of steady‐state gene expression profiles assessed by RNA‐seq. Percentage of variance accounted for by each principal component is indicated.BVolcano plot of differentially expressed genes comparing middle‐aged relative to young animals (FDR < 0.05, Wald test). 307 genes were up‐regulated (red) and 194 down‐regulated (blue).CVolcano plot of differentially expressed genes in the liver of aged versus young animals (FDR < 0.05, Wald test). 127 genes were up‐regulated (red) and 92 down‐regulated (blue). *n* = 3–4 biological replicates per age group.DPrincipal component analysis of nascent transcription assessed by tNET‐seq. PCA was performed with the normalized read counts (rlog transformation, DESeq2) in gene bodies of non‐overlapping, protein‐coding genes above 2 kb in size. The percentage of variance accounted for by each principal component is indicated.EVolcano plot of differentially transcribed genes (gene‐body Pol II density) in middle‐aged relative to young animals (FDR < 0.05, Wald test). 196 genes were up‐regulated (red) and 171 down‐regulated (blue).FVolcano plot of differentially transcribed genes (gene‐body Pol II density) in aged relative to young animals (FDR < 0.05, Wald test). 365 genes were up‐regulated (red) and 352 down‐regulated (blue). A, aged; MA, middle‐aged; Y, young. *n* = 3 biological replicates per age group.GCorrelation between changes in nascent (gene‐body Pol II density, tNET‐seq) and steady‐state transcription (RNA‐seq, generated of mice from our standing aging cohort at the MPI for Biology of Ageing) of aged versus young animals.HViolin and boxplots of changes in promoter accessibility (ATAC‐seq), nascent transcription (gene‐body Pol II density, tNET‐seq) and steady‐state transcription (RNA‐seq, generated of mice from our standing aging cohort at the MPI for Biology of Ageing) in aged versus young mice. Only genes present in all three datasets are included here (*n* = 2,698 genes). Box plots consist of the median (central line), the 25^th^ and 75^th^ percentiles (box) and the highest/lowest value within 1.5 * interquartile range of the box (whiskers). Promoter defined as TSS ± 200 bp.IVenn diagram of differentially transcribed (gene‐body Pol II density, tNET‐seq) and differentially expressed genes (RNA‐seq, generated of mice from our standing aging cohort at the MPI for Biology of Ageing) in aged versus young mice. Only significantly changed genes (FDR < 0.05, Wald test) were included. Up‐ or down‐regulated genes in both datasets are indicated in red and blue, respectively. Genes exhibiting divergent changes are indicated in grey. Data information: ATAC‐seq: young, *n* = 3; old, *n* = 4; all biological replicates; tNET‐seq: three biological replicates per age group.

To understand how genome accessibility changes on a local scale with age, we performed ATAC‐seq using liver tissue from independent biological replicates of each young (3‐month‐old) and aged (18‐month‐old) mice (Fig 1A; Appendix Fig S1). The generated ATAC‐seq datasets were of high quality and displayed the characteristic fragment size distribution (Appendix Fig S2A–D). As a first‐level analysis, we performed principal component analysis (PCA). PCA revealed that samples from the same age group (young or aged) formed two distinct clusters mostly distinguishable by the first two principal components, which explain more than 70% of the variance in the data (Appendix Fig S2E). Differential accessibility analysis revealed that only 8.43% of analyzed regions (4,691 out of 55,669) were differentially accessible with age (Fig 1B). Of these, 2,760 sites became more accessible, while 1,931 sites showed decreased accessibility with age. This slight trend toward an overall increased accessibility prompted us to further zoom into the differentially accessible sites by investigating their genomic location. We observed that sites with increased accessibility in the aged liver were on average closer to annotated TSSs compared with less accessible sites, suggesting an age‐related increase in the accessibility of promoter regions (Fig EV1A). Indeed, promoter regions and 5′ UTRs became more accessible with age, while the accessibility of distal intergenic and genic sites decreased (Fig 1C). In fact, the majority of regions (63.4%), for which we detected an increased accessibility, were located within annotated promoter regions (Fig 1D). In contrast, only 15.0% of regions with decreased accessibility in aged animals overlapped with known promoter regions (Fig 1D). This age‐related increase in chromatin accessibility at promoter regions was detectable both on a metagene (Fig 1E) and single‐gene level (Fig 1F) and could also be observed in a previously published MNase‐seq dataset (Appendix Fig S3; Bochkis *et al*, 2014). Furthermore, gene ontology (GO) enrichment analysis revealed that genes with more accessible promoters in aged liver tissue were involved in metabolic processes such as amino acid metabolism (Fig EV1B; Table EV1). In contrast, genes with decreased promoter accessibility with age were linked to nucleosome assembly and organization (Fig EV2C; Table EV2). Together, our results indicate that liver aging is accompanied by an increased chromatin accessibility at promoter regions.

### Aging has a modest effect on the transcriptional output

**Figure 2:**
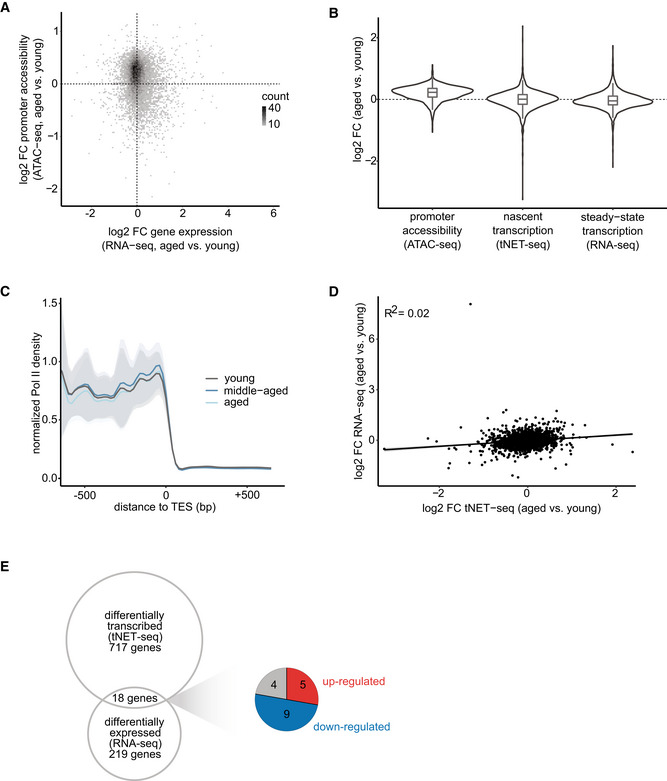
Aging has a subtle effect on transcriptional output in murine liver tissue AScatter plot of the log_2_‐fold change in gene expression (RNA‐seq) and promoter accessibility (ATAC‐seq) in aged versus young mice. Promoter region defined as TSS ± 200 bp.BViolin and boxplots of changes in promoter accessibility (ATAC‐seq), nascent transcription (gene body Pol II density, tNET‐seq), and steady‐state transcription (RNA‐seq) in aged versus young mice. Only genes present in all three datasets are included here (*n* = 2,693 genes). Promoter defined as TSS ± 200 bp. Box plots consist of the median (central line), the 25^th^ and 75^th^ percentiles (box), and the highest/lowest value within 1.5 * interquartile range of the box (whiskers).CMetagene profile showing normalized Pol II densities (tNET‐seq) at TESs. Read densities of merged biological replicates (*n* = 3) were normalized to 1× coverage. Solid lines represent mean values, and shading indicates the 95% confidence interval.DCorrelation between changes in nascent (gene body Pol II density, tNET‐seq) and steady‐state transcription (RNA‐seq) of aged versus young animals.EVenn diagram of differentially transcribed (gene body Pol II density, tNET‐seq) and differentially expressed genes (RNA‐seq) in aged versus young mice. Only significantly changed genes (FDR < 0.05, Wald test) were included. Up‐ or downregulated genes in both datasets are indicated in red and blue, respectively. Genes exhibiting divergent changes are indicated in gray. Data information: RNA‐seq: young, *n* = 4; middle‐aged, *n* = 4; aged, *n* = 3; all biological replicates; ATAC‐seq: young, *n* = 3; old, *n* = 4; all biological replicates; tNET‐seq: three biological replicates per age group.

Given the central role of promoter accessibility in transcription, we next asked whether the observed age‐related increase in promoter accessibility affects the transcriptional output. For this, we assessed steady‐state transcription using publicly available liver RNA‐seq data from the Tabula Muris Senis Consortium (Schaum *et al*, 2020). For a more detailed look into the temporal dynamics of gene expression changes with age, we exploited the availability of gene expression data from multiple age groups and included, in addition to young and old (3 and 18 months of age, respectively), also middle‐aged (12‐month‐old) mice (Fig 1A). Gene expression patterns in aged liver were clearly distinct from those of young and middle‐aged animals as observed by PCA (Fig EV2A). Differential expression analysis revealed that the expression of the majority of genes did not significantly change with age: 2.73% (501 out of 18,325) and 1.40% (219 out of 15,610) of analyzed genes were differentially expressed in middle‐aged and aged compared with young animals, respectively (Fig EV2B and C). Thus, gene expression in the liver appears to be relatively resistant to aging, consistent with observations from single‐cell RNA‐seq data of liver hepatocytes from the Tabula Muris Consortium (Tabula Muris Consortium, 2020).To directly assess the effect of promoter accessibility on the transcriptional output, we integrated our ATAC‐seq with the RNA‐seq data. Interestingly, the age‐related increase in promoter accessibility is not directly reflected in increased transcriptional output (Fig 2A). Of note, standard RNA‐seq measures steady‐state mRNA levels, which are determined by both synthesis and degradation rates. To exclude the contribution of mRNA degradation, we next focused exclusively on nascent transcription.

To investigate age‐induced changes in nascent transcription on a genome‐wide level, we employed native elongating transcript sequencing (NET‐seq) (Mayer *et al*, 2015). NET‐seq quantitatively maps the position of transcriptionally engaged Pol II with single‐nucleotide resolution and strand specificity. Importantly, NET‐seq does not require the previous labeling of mRNA and, thus, is ideally suited for measuring nascent transcription in tissues. In brief, actively transcribing Pol II together with the nascent RNA is quantitatively purified from cells by cellular fractionation. To prevent run‐on transcription, fractionation is performed in the presence of the potent Pol II inhibitor α‐amanitin, which prevents NTP recognition and catalysis by the Pol II trigger loop (Brueckner & Cramer, 2008; Kaplan *et al*, 2008). Then, the isolated nascent RNA is fragmented, converted into cDNA, and processed into a sequencing library employing a minimal number of PCR cycles. The library preparation method is designed in a way that upon random fragmentation of the nascent transcripts, only the 3′ ends of nascent transcripts carrying a free hydroxy‐group are sequenced. This reveals the position of transcriptionally engaged Pol II at nucleotide resolution.

For application in murine liver tissue, we modified the protocol to include efficient tissue homogenization and nuclei isolation in one single step. We named this new, modified version of the original protocol tissue NET‐seq (tNET‐seq). We performed tNET‐seq using freshly isolated liver tissue from three independent biological replicates from young (3‐month‐old), middle‐aged (12‐month‐old), and aged (18‐month‐old) mice. These age groups match those of the RNA‐seq data, enabling direct integration of the two datasets. tNET‐seq libraries showed high reproducibility among biological replicates (Appendix Fig S4A and B), indicating the high robustness of the approach.

Using tNET‐seq, we assessed how nascent transcription is affected by age. For this, we stringently defined a set of protein‐coding genes that do not overlap with other transcription units within 2.5 kb of the TSS and TES and are longer than 2 kb (*n* = 12,460 genes). We further included only genes with sufficient coverage (RPKM > 1, *n* = 3,280 genes). Using these genes, hereafter referred to as tNET genes, we assessed Pol II density at TES as a proxy for nascent transcriptional output. Consistent with the modest changes we observed in steady‐state expression levels, the nascent transcriptional output was also only mildly affected with age (Fig 2B and C). Yet, there were clear differences between the nascent transcriptome profiles of young and aged liver, assessed by PCA (Fig EV2D). To further dissect these age‐related changes in nascent transcription, we quantified the nascent transcript levels by assessing Pol II density within gene bodies. Compared with young animals, we found 11% (367 out of 3,278) and 22% (717 out of 3,278) of genes to be differentially transcribed in middle‐aged and aged liver, respectively (Fig EV2E and F). There was an equal distribution between up‐ and downregulated nascent transcripts, highlighting the fact that there is no global unidirectional shift (i.e., increase or decrease) in the nascent transcription with age. Overall, these results demonstrate that while promoter accessibility is a requirement for transcription, its increased accessibility in the aging liver does not automatically result in elevated transcriptional output.

### Age‐related changes in nascent transcription are not directly mirrored in steady‐state mRNA levels

To link nascent with steady‐state transcription, we directly compared tNET‐seq and RNA‐seq data sets. The moderate positive correlation between the normalized signal indicated an overall agreement between the two data sets (Appendix Fig S5). However, we observed only a weak positive correlation between age‐related changes in nascent and steady‐state transcription (Fig 2D). In fact, only 18 genes were found to be both differentially transcribed (tNET‐seq) and differentially expressed (RNA‐seq) with age (Fig 2E). A similar effect was seen when we compared RNA‐seq of young and old liver generated from an aging mouse cohort from our animal house and thus more comparable to the (t)NET‐seq data (Fig EV2F–I). Together, these results demonstrate that age‐related changes in nascent transcription do not necessarily reflect the steady‐state mRNA levels and highlight the important contribution of post‐transcriptional regulatory processes, such as mRNA degradation, to steady‐state mRNA levels.

### Age‐related changes in nascent transcription lack a clear signature

**Figure EV3:**
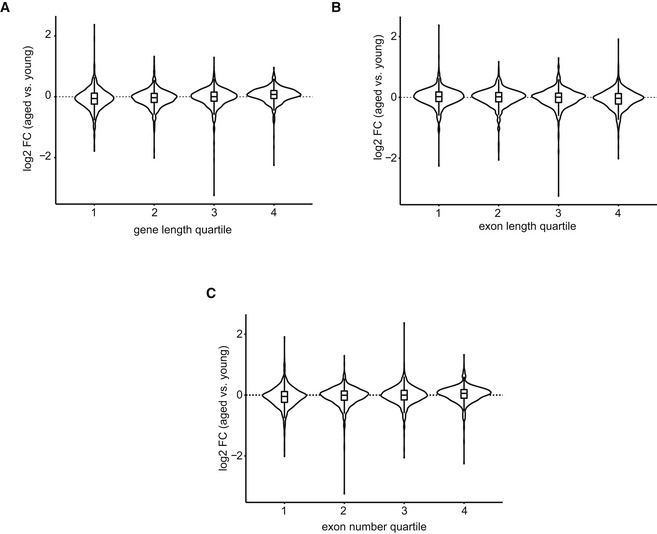
Potential transcript features and impact on nascent transcription upon aging A–CChanges in nascent transcription (gene‐body Pol II density, tNET‐seq) in aged versus young animals in relationship to (A) gene length, (B) median exon length or (C) exon number per gene. The gene length was calculated as the total exonic length after reducing a gene's exons to a non‐overlapping set. Number of genes per quartile: 819. Box plots consist of the median (central line), the 25^th^ and 75^th^ percentiles (box) and the highest/lowest value within 1.5 * interquartile range of the box (whiskers). *n* = 3 biological replicates per age group.

Considering the limited number of genes convergently changing on nascent and steady‐state level, we next focused our attention exclusively on the nascent transcription and investigated whether differentially transcribed genes exhibit a specific signature in their gene features. We found no correlation between age‐related changes in nascent transcription and gene length (Fig EV3A), exon length (Fig EV3B), or exon number per gene (Fig EV3C).

**Figure 3:**
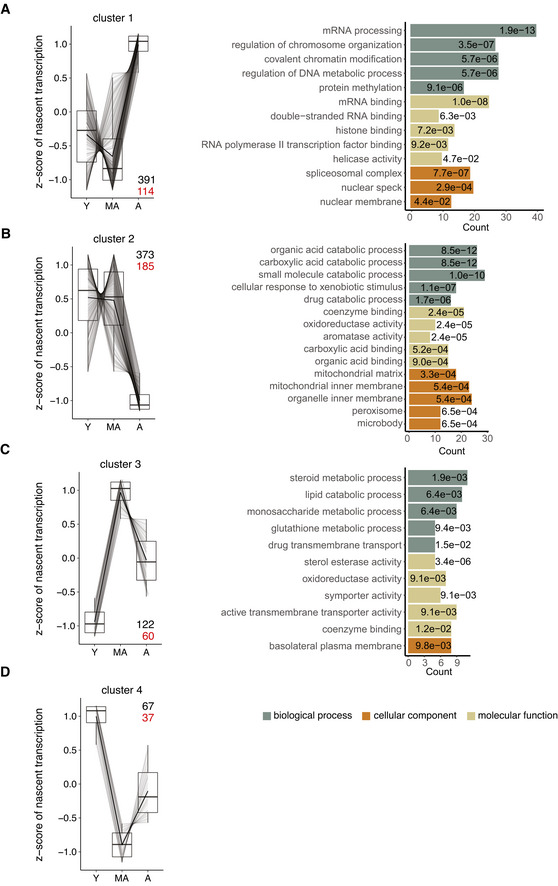
Trajectory analysis of differentially transcribed genes in aged versus young mice A–DAnalysis resulted in four distinct gene clusters (*n* = 953 genes, likelihood ratio test, DESeq2). In each cluster, the total number of genes and the number of “aging genes” are designated in black and red, respectively. Nascent transcript levels are reported in the parallel coordinate plot on the left as *z*‐scores of gene body Pol II density. GO term analysis for each gene cluster is depicted on the right. Only the top five enriched GO terms ranked by adjusted *P*‐value (Benjamini‐Hochberg (BH) procedure, FDR < 0.05) are displayed. BH‐adjusted *P*‐values are reported for each GO term inside the respective bar. A—aged; MA—middle‐aged; Y—young. *n* = 3 biological replicates per age group. Box plots consist of the median (central line), the 25^th^ and 75^th^ percentiles (box) and the highest/lowest value within 1.5 * interquartile range of the box (whiskers).

To dissect temporal trajectories of changes in nascent transcription, we performed differential testing using a likelihood ratio test. Considering young, middle‐aged, and aged animals, we found 29% of genes (953 out of 3,278) to be differentially transcribed. Trajectory analysis of these differentially transcribed genes resulted in four distinct gene clusters with similar patterns and distinct functional enrichment (Fig 3): Genes in cluster 1 showed an age‐related increase in nascent transcription and were linked to mRNA processing and splicing (Fig 3A). Genes in clusters 2 and 3 exhibited an overall decrease and increase, respectively, and were linked to metabolic processes (Fig 3B and C). While cluster 2 was functionally linked to xenobiotic and carboxylic acid catabolic processes, genes in cluster 3 were involved in lipid, carbohydrate, or glutathione metabolic processes. This highlights the diversity of age‐related, metabolic changes in the highly metabolic liver tissue. The smallest gene cluster (cluster 4) contained very few genes (Fig 3D), which made it impossible to identify gene ontology enrichments. To understand the importance of these differentially transcribed genes in the context of murine aging, we cross‐referenced them to a recently published resource of “global aging genes” based on single‐cell RNA‐seq data from the Tabula Muris Senis Consortium (Zhang *et al*, 2021). A large fraction of the hepatocyte‐specific “aging genes” defined by the Tabula Muris Senis Consortium (45%, 1,158 out of 2,569) was present in our tNET‐seq dataset that we used for differential transcription testing and trajectory analysis. Of these hepatocyte‐specific “aging genes,” we found 34% (396 out of 1,158) to be differentially transcribed with age in our tNET‐seq dataset. These differentially transcribed “aging genes” were not enriched in any particular cluster suggesting no common pattern in the changes of their nascent transcription (Fig 3; Tables EV3–EV6). Taken together, these analyses reveal distinct age‐related trajectories in the nascent transcriptome with a large fraction of the “aging genes” previously defined by the Tabula Muris Senis Consortium as being altered. Yet, the changes in nascent transcription were not congruent and unidirectional, suggesting that nascent transcription alone does not control the coordinated global aging behavior at tissue and organismal level reported earlier. This corroborates the modest correlation between tNET‐seq and RNA‐seq we observed (Fig 2D), implying that the other processes, such as mRNA processing and degradation, might shape the coordinated gene expression signature detected within and across tissues.

### Promoter‐proximal Pol II pausing decreased with age

**Figure 4:**
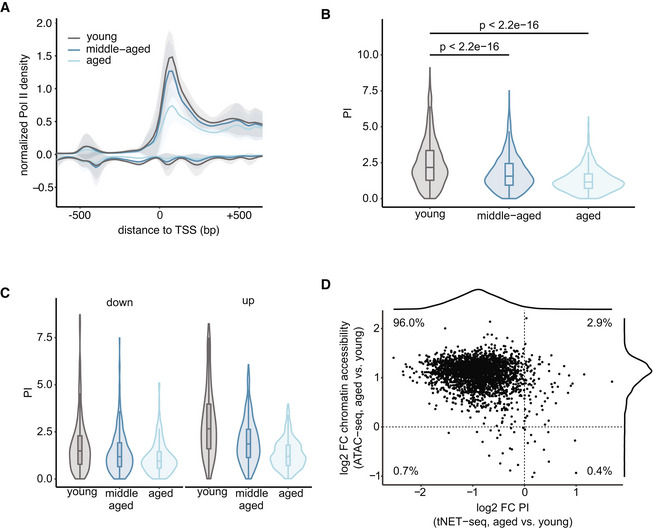
Promoter‐proximal Pol II pausing decreases with age in murine liver AMetagene profile of normalized Pol II densities (tNET‐seq) at the promoter‐proximal region of all genes (*n* = 3,280 genes) included in the analysis. Read densities of merged biological replicates (*n* = 3) were normalized to 1× coverage. Solid lines represent mean values, and shading indicates the 95% confidence interval.BViolin and box plots of PI values in young, middle‐aged, and aged animals. *P*‐values were calculated using a two‐sided Wilcoxon rank‐sum test. (*n* = 2,650 genes). Box plots consist of the median (central line), the 25^th^ and 75^th^ percentiles (box) and the highest/lowest value within 1.5 * interquartile range of the box (whiskers).CViolin and box plots showing the PI in young, middle‐aged, and aged animals. Only genes found to be differentially transcribed in aged versus young mice are included and grouped by the direction of change (downregulated, *n* = 267 genes, left panel; upregulated, *n* = 297 genes, right panel). Box plots consist of the median (central line), the 25^th^ and 75^th^ percentiles (box) and the highest/lowest value within 1.5 * interquartile range of the box (whiskers).DScatter plot of the change in PI (tNET‐seq) and promoter accessibility (ATAC‐seq) in aged versus young mice. Data information: Promoter region defined as TSS ± 200 bp. ATAC‐seq: young, *n* = 3; old, *n* = 4; all biological replicates; tNET‐seq: three biological replicates per age group.

**Figure EV4:**
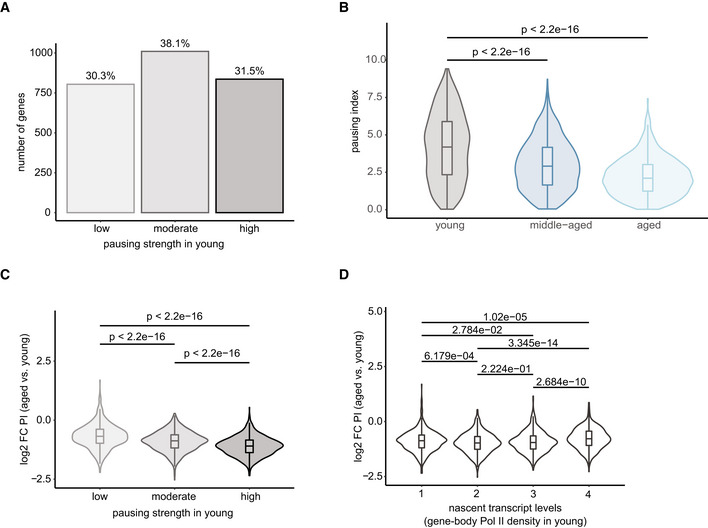
RNA Pol II pausing characteristics ABar plot of PIs quantifying the extent of promoter‐proximal pausing in young mice. The 2,650 genes were divided into three groups based on the PI in young animals: highly paused (PI ≥ 3; *n* = 836), moderately paused (1.5 ≤ PI < 3; *n* = 1,010), and lowly paused (PI < 1.5; *n* = 804).BViolin and box plots of PI values (based on TSS +20 to TSS +100 bp) in young, middle‐aged and aged animals. *P*‐values were calculated using a two‐sided Wilcoxon rank‐sum test (*n* = 1.289 genes). Box plots consist of the median (central line), the 25^th^ and 75^th^ percentiles (box) and the highest/lowest value within 1.5 * interquartile range of the box (whiskers).CViolin and box plot of change in PI of aged versus young liver. Genes were grouped by PI as in (A).DViolin and box plots of PI change in aged versus young animals. Genes were divided into four equal‐sized groups based on their level of nascent transcription (gene‐body Pol II density) in the liver of young mice (*n* = 2,650 genes). Box plots consist of the median (central line), the 25^th^ and 75^th^ percentiles (box) and the highest/lowest value within 1.5 * interquartile range of the box (whiskers). *P*‐values were calculated using a two‐sided Wilcoxon rank‐sum test. Data information: *n* = 3 biological replicates per age group.

The discrepancy between the increased promoter accessibility, the overall modest change in transcriptional output with age, and the lack of a clear signature in terms of common functionalities of differentially transcribed genes led us to focus on the initial stages of transcription at the promoter region, which is where much of the transcriptional regulation occurs. (t)NET‐seq is a powerful approach for investigating promoter‐proximal Pol II pausing, which is a major regulatory step of transcription. To quantify promoter‐proximal pausing, we calculated the pausing index (PI), which is the ratio of the average Pol II density in the promoter region (defined here as TSS ± 200 bp, for consistency with ATAC‐seq analysis) to that in the gene body (defined here as TSS +200 bp to TES −200 bp). The PI provides a measure of the magnitude of promoter‐proximal Pol II density relative to that in the gene body. Based on the PI values in young animals, we classified genes into three pausing categories with high (PI ≥ 3), moderate (1.5 < PI < 3), or low (PI ≤ 1.5) pausing. 69.7% of the analyzed genes were either highly or moderately paused in young animals (Fig EV4A). This corroborates promoter‐proximal pausing as a widespread phenomenon in the liver, consistent with previous reports (Adelman & Lis, 2012) in other organisms and cell types. Importantly, a more narrowly‐defined promoter‐proximal pausing site as usually used in the literature (Adelman & Lis, 2012) gave very similar results (Fig EV4B). To investigate alterations in promoter‐proximal Pol II pausing with age, we generated metagene profiles of a 1‐kb region around the genes' TSSs (Fig 4A). Notably, we observed a progressive age‐related decrease in promoter‐proximal Pol II pausing (Fig 4A). This decrease, however, was not accompanied by changes in Pol II density in the immediate 5′ gene bodies (Fig 4A) or at the TES as a proxy for transcriptional output (Fig 2C). The age‐related decrease in promoter‐proximal pausing is also reflected in a progressive decrease in PI (Fig 4B). Interestingly, highly paused genes (PI > 3 in young mice) experienced the highest decrease in promoter‐proximal Pol II pausing with age (Fig EV4C). Changes in the PI depend on both changes in the numerator (promoter‐proximal Pol II) and denominator (gene body Pol II). Therefore, we considered the possibility that the age‐related decrease in PI may be related to the levels of nascent transcription. Hence, we grouped the gene set into equal‐sized groups based on their level of nascent transcription in young mice (Fig EV4D). The fold changes in PI between all groups were similar, indicating that the age‐related decrease in PI occurs irrespective of the gene's expression level. This was further confirmed by the observation that differentially transcribed genes consistently exhibited a lower PI in aged animals (Fig 4C). This is true even for the downregulated genes, corroborating that the observed effect was specific to the promoter‐proximal region (numerator of PI) and could not be explained solely by changes in nascent transcription (denominator of PI). Overall, these findings reveal an age‐related decrease in promoter‐proximal Pol II pausing affecting the majority of the analyzed genes.

The local chromatin landscape at promoter regions directly modulates transcriptional regulation and progression of Pol II by regulating the accessibility of the region. To investigate the relationship between promoter accessibility and promoter‐proximal pausing, we integrated the ATAC‐seq and tNET‐seq datasets. The majority of investigated promoters (96.0%) exhibited an increased chromatin accessibility with a concomitant decrease in promoter‐proximal Pol II pausing (Fig 4D). In light of the modest age‐related changes in transcriptional output (Fig 2B), this points toward a compensatory mechanism, in which alterations at the step of promoter‐proximal pausing might counteract the increased accessibility of the promoter regions, thereby maintaining transcriptional fidelity.

### Aging affects the stability of the Pol II pausing complex

How can the age‐related decrease in promoter‐proximal Pol II pausing be explained? Focusing solely on the transcription process, we can envision three possible scenarios: A decrease in promoter‐proximal Pol II pausing can be a consequence of a decreased initiation rate, a decreased duration of pausing, or a combination of both.

**Figure 5:**
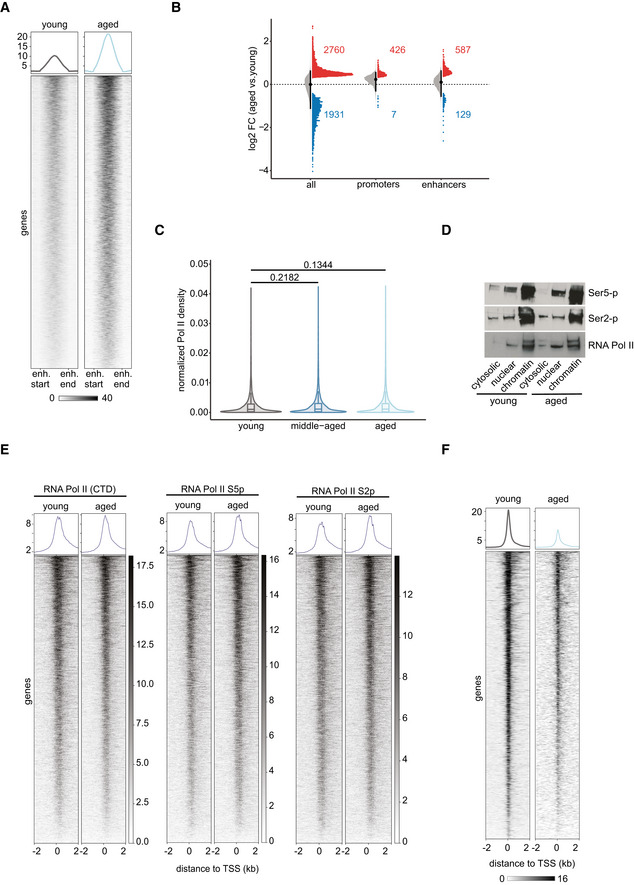
Aging affects the stability of the Pol II pausing complex rather than transcription initiation AHeatmap and intensity profiles of promoter accessibility over active enhancers in liver (*n* = 8,855). Read densities of merged biological replicates (*n* = 3–4) were normalized to 1× coverage.BRaincloud plots of changes in accessibility in aged versus young liver assessed by ATAC‐seq. Raincloud plots are hybrid plots. Here, the violin and boxplots visualize the log2‐fold change for all accessible genomic sites (“all”) or accessible sites overlapping promoters of tNET genes (“promoters”) or active enhancers in murine liver (“enhancers”). The dot plot highlights regions significantly changed with age in each category (FDR < 5%). The numbers in the plot denote significantly up‐ or downregulated regions in each category.CViolin and boxplots of Pol II density at enhancer regions (*n* = 8,144) as a means of quantifying eRNA production. *P*‐values were calculated using a two‐sided Wilcoxon rank‐sum test. Box plots consist of the median (central line), the 25^th^ and 75^th^ percentiles (box) and the highest/lowest value within 1.5 * interquartile range of the box (whiskers).DWestern blots of cellular fractionations of young and aged liver samples probed with the indicated antibodies.EHeatmaps and average intensity profiles of RNA Pol II and its S2 and S5 phosphorylated forms in young and aged animals.FHeatmaps and average intensity profiles of SPT4 ChIP‐seq signal at TSSs of tNET genes in young and aged animals. Data information: *n* = 3–4 biological replicates per age group for tNET‐seq, ATAC‐seq and CUT&RUN; *n* = 2 for ChIP‐seq.

**Figure EV5:**
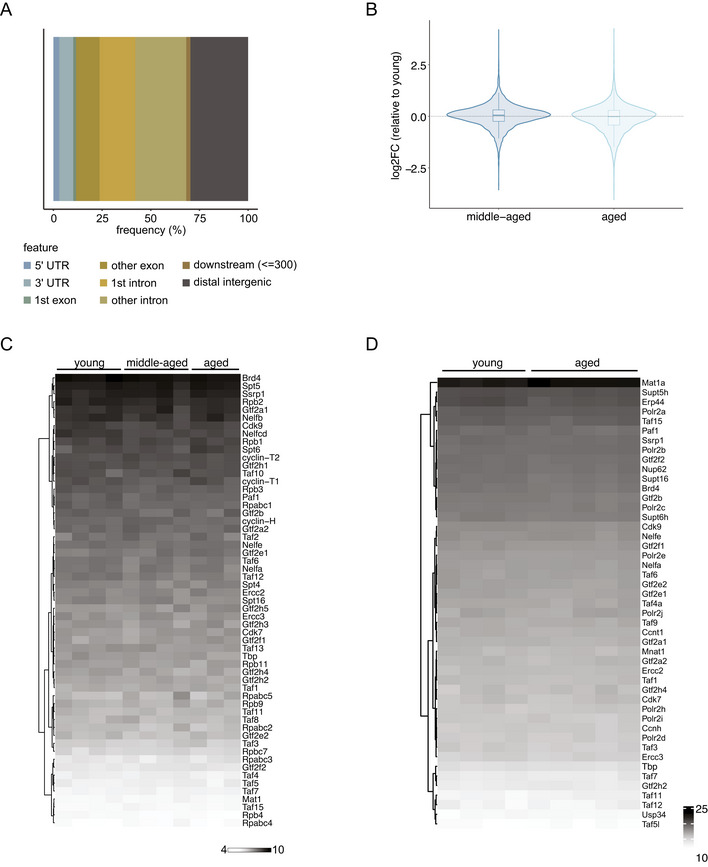
Enhancers and TF abundance AGenomic distribution of identified active enhancers.BViolin and boxplots of log2‐fold changes in Pol II density at enhancer regions of middle‐aged and aged animals relative to young ones (*n* = 8,144). Box plots consist of the median (central line), the 25^th^ and 75^th^ percentiles (box) and the highest/lowest value within 1.5 * interquartile range of the box (whiskers).CHeatmap of steady‐state mRNA levels (RNA‐seq) of relevant transcription regulators in young, middle‐aged and aged mice. Normalized read counts (rlog transformation, DESeq2) are reported.DHeatmap of protein abundance of relevant transcription regulators in young and aged mice assessed by mass spectrometry. Normalized TMT reporter intensities (vsn‐normalization, limma) are reported. Data information: *n* = 3–4 biological replicates per age group for RNA‐seq and tNET‐seq; *n* = 4–5 for proteomics as indicated in the heatmaps.

Alterations in transcription initiation would be detected as decreased promoter‐proximal Pol II pausing. (t)NET‐seq does not allow for directly quantifying initiating Pol II, since the nascent RNA needs to be at least 20 bp long for adapter ligation and unambiguous read alignment to the genome. Therefore, we focused on enhancer regions instead, which regulate transcription initiation at multiple levels (Beagrie & Pombo, 2016). For identifying active enhancers in murine liver, we integrated our ATAC‐seq data with publicly available histone ChIP‐seq data from liver tissue of young (3 months), middle‐aged (12 months), and aged (29 months) mice (Benayoun *et al*, 2019). This resulted in the identification of 8,855 high‐confidence, putative enhancers, which were present in all three age groups. The majority of these enhancers are located in intronic regions (Fig EV5A), consistent with a recent report highlighting the predominantly intronic location of tissue‐specific enhancers (Borsari *et al*, 2021). Having identified these liver‐specific enhancers, we then assessed enhancer activity using chromatin accessibility as a proxy. We observed an increased enhancer accessibility in aged compared with young animals (Fig 5A). In fact, 21.3% (587 out of 2,760) of the genomic regions that were more accessible with age were located within enhancer regions (Fig 5B). In contrast, only 6.7% (129 out of 1,931) of sites that exhibited a significant decrease in accessibility with age were found within enhancer regions (Fig 5B).

This trend of more enhancer sites becoming more accessible with age compared to those with decreased accessibility mirrors the accessibility changes at promoters (Fig 1E). As a second proxy for enhancer activity, we quantified enhancer RNA transcription, since eRNA production and enhancer activity have been reported to highly correlate on a genome‐wide scale. (t)NET‐seq captures nascent RNAs, including short‐lived RNA species like eRNAs, which are not detected with sufficient coverage by RNA‐seq. We quantified eRNA production by assessing Pol II levels at the identified enhancer regions. We observed no age‐related change in eRNA production (Figs 5C and EV5B). Overall, our data point toward unaltered or increased, but not decreased initiation rates. As decreased promoter‐proximal pausing occurs globally, an overall decline in initiation should be visible at the level of CTD phosphorylation. In line with our analysis on enhancer activity, we did not observe any change in the level of Ser5 nor Ser2 phosphorylation (Fig 5D). To more directly test potential changes in RNA Pol II loading, we performed CUT&RUN analysis of RNA Pol II and its S2 and S5 phosphorylated form (Fig 5E). In line with all previous analysis performed, we did not observe any change in RNA Pol II occupancy, nor a change in S2 and S5 phosphorylation, further supporting the notion that RNA Pol II loading is not globally affected in aged liver. Taken together, these data in combination with the overall increase in promoter accessibility strongly suggest that transcription initiation is not compromised in aged livers but is likely enhanced.

The second possible explanation for the age‐related decrease in promoter‐proximal Pol II pausing is an age‐related decrease in Pol II dwell time. We first assessed the expression levels of important transcriptional regulators involved in the early stages of transcription. We observed no change in the expression of these factors with age—neither on the level of steady‐state mRNA nor on protein level (Fig EV5C and D). However, while the expression of these transcription regulators was not affected by age, their recruitment to chromatin might have been altered. To assess this, we performed ChIP‐seq for SPT4, a component of the pause‐inducing factor DSIF. We observed a decreased occupancy of SPT4 at TSSs of tNET genes in aged animals (Fig 5F). Thus, while their expression does not seem to be affected with age, the recruitment and binding of important pausing factors to chromatin might be altered, resulting in changes in promoter‐proximal Pol II pausing.

**Figure 6:**
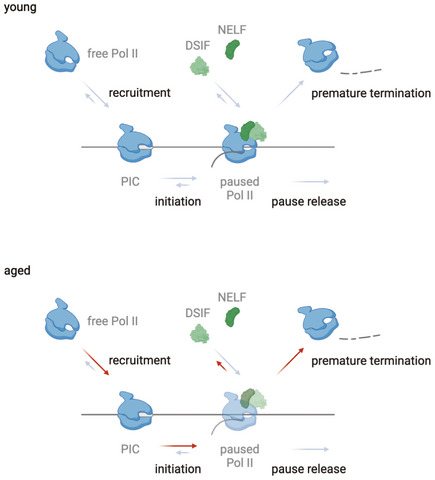
Proposed model for the age‐related changes that occur in the liver Increased chromatin accessibility and indirect assessment of initiation suggest that this step is not affected, or rather increased as indicated by the red arrows. On the contrary, decreased pausing in the promoter‐proximal region and lower recruitment of SPT4 suggest that the pausing complex is less stable upon aging, resulting in premature termination of transcription (indicated by red arrow).

This is consistent with the decreased level of promoter‐proximal Pol II pausing we observed using tNET‐seq (Fig 4A and B). These results suggest that the age‐related decrease in promoter‐proximal Pol II pausing might be explained by an altered stability of the pausing complex in aged animals. It is tempting to speculate that the dissociation of Pol II from chromatin at this early stage of transcription might antagonize the effect of the age‐related increase in promoter accessibility and, thus, a potential increase in Pol II recruitment and transcription initiation. Overall, a potential increase in initiation and a concomitant decrease in stability of the pausing complex would lead to an unchanged transcriptional output (Fig 6).

## Discussion

The rapid development of omics technologies in recent years has significantly advanced our understanding of the epigenome and its role in transcriptional regulation. Using freshly isolated liver tissue from young, middle‐aged, and aged mice, we provide a comprehensive analysis of age‐related changes in the local chromatin landscape and the nascent transcriptome. The combination of multiple genome‐wide sequencing techniques (tNET‐seq, ATAC‐seq, RNA‐seq, and ChIP‐seq) creates a framework for investigating the connection between chromatin and transcriptional regulation upon aging.

In recent years, several studies have interrogated age‐related changes in the chromatin landscape of different tissues and cell systems. Chromatin accessibility analysis of aged CD8^+^ T‐cells from human donors revealed a decrease in promoter accessibility (Moskowitz *et al*, 2017; Ucar *et al*, 2017). Recently, an overall more compacted chromatin architecture has also been observed in aged murine neutrophils (Lu *et al*, 2021) and mesenchymal stem cells (Pouikli *et al*, 2021). Contrasting these observations of decreased chromatin accessibility, a study reports no major changes in accessibility in aged murine B precursor cells (Koohy *et al*, 2018). Thus, it is likely that age‐related alterations in chromatin accessibility are highly tissue‐ and cell‐type specific, which may, at least in part, explain these contrasting observations. We find that in murine liver tissue, a specific fraction of the genome undergoes age‐related changes. Particularly, promoter regions gain accessibility. Importantly, we do not observe changes in the expression of histone genes, indicating that the increase in promoter accessibility is not simply a consequence of decreased nucleosomes. In support of this, no global changes in nucleosome occupancy (assessed using H3 occupancy as a proxy) have been reported in aged murine liver tissue; only at a subset of loci either increased or decreased occupancy was observed (Chen *et al*, 2020). Previous work also highlighted that aging in the liver is not accompanied by major changes in cell‐type composition (preprint: Nikopoulou *et al*, 2021).

Despite the increase in promoter accessibility, we observe only modest effects on both the nascent and steady‐state transcriptome with age. This highlights that the expression levels of most genes are generally preserved in aged liver, consistent with the notion that liver tissue seems to be more refractory to aging (Zhang *et al*, 2021) as assessed by an aging score based on scRNA‐seq data from the Tabula Muris Consortium (Tabula Muris Consortium, 2020). Hepatocytes from different age groups exhibited similar aging scores, suggesting that their transcriptional programs are marginally affected by age (Zhang *et al*, 2021). This is in contrast, for instance, to immune and stem cells that exhibited a stronger increase in aging scores, reflecting the higher turnover rates of these cell types. Intriguingly, we observed only a small overlap between differentially expressed and differentially transcribed genes upon aging, with more widespread changes identified in the nascent transcriptome. A similar discordance has been observed in total versus nuclear RNA‐seq analyses of young and aged murine B precursor cells (Koohy *et al*, 2018). Thus, changes in the nascent transcriptome might be buffered through post‐transcriptional mechanisms affecting RNA stability. This is consistent with recent results analyzing single‐cell RNA‐ and ATAC‐seq data of aged murine liver, which also highlighted the important contribution of post‐transcriptional processes (preprint: Nikopoulou *et al*, 2021).

Despite the modest age‐related effects on transcriptional output, we observed a strong decrease in promoter‐proximal Pol II pausing. How can this age‐related decrease in promoter‐proximal Pol II pausing be explained? We provide data demonstrating reduced recruitment of the DSIF subunit SPT4 to the promoter‐proximal region, suggesting that the pausing complex is less stable in aged hepatocytes. However, we cannot distinguish between a reduced recruitment to or an increased dissociation from chromatin. How DSIF is recruited to Pol II remains enigmatic. A recent mass spectrometry analysis revealed that the proto‐oncogene MYC recruits the second DSIF subunit, SPT5, to promoters, thereby promoting the assembly of SPT5 with Pol II and controlling the processivity of Pol II elongation complexes (Baluapuri *et al*, 2019). Furthermore, MYC also regulates pause release by recruiting the P‐TEFb subunit CDK9 to promoters (Rahl *et al*, 2010). Considering the link of MYC to inflammation and cancer (Greten & Grivennikov, 2019), it would be interesting to further explore its role in the context of aging and promoter‐proximal Pol II pausing. Besides an altered recruitment of the pausing factors, aging might affect the dissociation of the pausing complex from chromatin. SPT5 depletion using RNAi in Drosophila S2 cells led to a loss of promoter‐proximal Pol II, without a release into productive elongation, suggesting increased levels of promoter‐proximal transcription termination (Henriques *et al*, 2018). This mirrors effects observed in NELF‐depleted cells, where turnover of promoter‐proximal Pol II was more rapid (Gilchrist *et al*, 2010; Henriques *et al*, 2013; Shao & Zeitlinger, 2017). Thus, DSIF and NELF are central to regulating the stability of paused Pol II. Recent studies using rapid inducible protein depletion have provided an even more fine‐grained view of the roles of DSIF and NELF. Acute SPT5 depletion led to destabilization and degradation of promoter‐proximal Pol II (Aoi *et al*, 2021; Hu *et al*, 2021). In contrast, acute NELF depletion triggered premature termination of promoter‐proximally paused Pol II (Aoi *et al*, 2020). Both of these distinct pathways result in the removal of Pol II from chromatin. These lines of evidence suggest that our results could be explained by a reduced stability of paused Pol II with age. It is currently actively debated on how much of the paused Pol II complex proceeds into productive elongation versus promoter‐proximal termination (Core & Adelman, 2019). Our results suggest an age‐related shift of the balance toward promoter‐proximal termination. Further experimentation will elucidate the magnitude with which these contribute to the observed age‐related decrease in promoter‐proximal Pol II pausing. Of particular, interest is the Integrator complex, whose RNA endonuclease activity has been implicated in promoter‐proximal transcription termination (Skaar *et al*, 2015; Elrod *et al*, 2019). Notably, both NELF and DSIF can associate with the Integrator complex (Yamamoto *et al*, 2014).

Overall, to explain the age‐related decrease in promoter‐proximal Pol II pausing, we propose the following model (Fig 6): With age, chromatin accessibility at promoters of protein‐coding genes increases, while recruitment of polymerase to promoters is not impacted. However, promoter‐proximal pausing decreases progressively with age and the decreased recruitment of SPT4 suggests that aging is accompanied by an increase in premature termination. Future work will have to address the upstream events that lead to these age‐related changes, whether they represent a compensation for the increase in promoter accessibility in order to maintain a balanced transcriptome, altered signaling events or changes in the efficiency of chromatin remodeling.

## Materials and Methods

### Reagents and Tools table

**Table:** 

Reagent/resource	Reference or source	Identifier or catalog number
**Experimental models**		
C57BL/6N (M. musculus)	Charles River Germany, bred in house (Transgenic Core Facility of MPI for Biology of Ageing, Cologne, Germany)	strain code: 027
**Antibodies**		
Rabbit‐anti SPT4	Cell Signaling Technology	64828
normal rabbit IgG	Cell Signaling Technology	2729S
anti‐rabbit IgG, HRP‐coupled	Cell Signaling Technology	7074S
RNA Pol II	Abcam	ab817
RNA Pol II Ser2 phospho	Abcam	ab5095
RNA Pol II Ser5 phsopho	Abcam	ab5408
**Oligonucleotides and sequence‐based reagents**		
ATAC‐seq PCR primers	Buenrostro *et al* (2013)	
tNET‐seq olignucleotide sequences	Mayer *et al* (2015)	
tNET depletion oligonucleotides	Mylonas and Tessarz (2018)	
ChIP‐seq / CUT&Run library prep primers	Bioo Scientific, NEXTflex	514112
**Chemicals, enzymes and other reagents**		
Invitrogen™ Digitonin	Thermo Fischer	BN2006
DNase I (RNase‐free, 20,000 U/ml)	NEB	M0303
NEBNext® High‐Fidelity 2× PCR Master Mix	NEB	M0541
TD buffer and Tn5 transposase	Illumina	20034197
SYBR™ Green I	Thermo Fischer	S7563
α‐amanitin	Sigma‐Aldrich	A2263‐1MG
Halt™ protease inhibitor cocktail (100×)	Thermo Fischer	Cat # 78429
Recombinant RNasin Ribonuclease Inhibitor	Promega	N2511
T4 DNA polymerase	NEB	M0203S
Klenow fragment (3′ ➔ 5′ exo‐)	NEB	M0212S
T4 Polynucleotide Kinase (PNK)	NEB	M0201S
Quick Ligation™ Kit	NEB	M2200S
Deoxynucleotide Solution Set	NEB	N0446S
RNase A	Thermo Fischer	EN0531
proteinase K	Thermo Fischer	EO0491
AMPure XP beads	Beckman Coulter	A63881
pAG‐Mnase (CUTANA for CUT&Run)	Epicypher	SKU 15‐1016
Digitonin (5%)	Thermo Fischer	BN2006
BioMag®Plus Concanavalin A	Polysciences	86057
**Software**		
FastQC	https://www.bioinformatics.babraham.ac.uk/projects/fastqc/	v 0.11.5
cutadapt	Martin (2011)	v 1.13
STAR	Dobin *et al* (2012)	v. 2.7.3a
Bowtie2	Langmead & Salzberg (2012)	v 2.4.1
PicardTools	http://broadinstitute.github.io/picard/	v 2.21.4
samtools	Li *et al* (2009)	v 1.10
Rsamtools	https://bioconductor.org/packages/release/bioc/html/Rsamtools.html	v. 2.2.3
ATACseqQC	Ou *et al* (2018)	v. 1.14.4
MACS2	preprint: Gaspar (2018)	v. 2.2.7
ChIPseeker	Yu *et al* (2015)	v. 1.26.2
ChIPpeakAnno	Zhu *et al* (2010)	v 3.24.2
Rsubread	Liao *et al* (2019)	v. 2.0.1
DESeq2	Love *et al* (2014)	v. 1.26.0
bedtools	Quinlan & Hall (2010)	v. 2.29.2
DEGreport	Pantano (2019)	v. 1.26.0
clusterProfiler	Yu *et al* (2012)	v. 3.14.3
Deeptools	Ramírez *et al* (2014)	v. 3.5.1
Integrative Genomics Viewer	Robinson *et al* (2011)	v. 2.8.0
SEACR	Meers *et al* (2019)	v. 1.3
R		v. 3.6.3
python		v. 3.9.0
**Other**		
Nuclei Isolation Kit	Sigma‐Aldrich	NUC101‐1KT
DNA Clean & Concentrator™‐5 kit	Zymo Research	D4013
Qubit dsDNA HS assay kit	Thermo Fischer	Q32851
Nucleospin Gel and PCR Mini Clean up kit	Macherey Nagel	740609.5

### Methods and Protocols

#### Mouse husbandry

Animals were bred and housed in the mouse facility of the Max Planck Institute for Biology of Ageing. Experimental procedures were approved by the State Office North Rhine‐Westphalia, Germany (LANUV), and are in accordance with institutional and national guidelines. Young (3‐month‐old), middle‐aged (12‐month‐old), and aged (18‐month‐old) male C57BL/6N mice were used. Mice were provided with *ad libitum* standard rodent diet and water. We used physiologically healthy individuals that show no signs of fibrosis, inflammation, and/or pathological steatosis (Appendix Fig S1) and were comparable to animals used in previous publications (preprint: Nikopoulou *et al*, 2021).

#### H&E staining

For IHC stainings, sections of paraffin‐embedded samples from two young and two old mice were deparaffinized by immersion of the slides into the following buffers; 20 min in Xylol, 2 min 100% EtOH, 2 min 96% EtOH, 75% EtOH and 1× PBS and washed three times with H_2_O for 5 min each. Following deparaffinization, slides with tissues washed with distilled and tapped water and stained with Ηematoxylin for 5 min, followed by five washes in tapped water and staining with Eosin Y for 3 min, followed by three washes with tap water, dehydration and mounting in Entellan. Images were taken using a Nikon Eclipse Ci microscope, with a color camera.

#### ATAC‐seq library preparation

ATAC‐seq libraries were prepared following the Omni‐ATAC protocol (Corces *et al*, 2017) using liver tissue of three young and four aged biological replicates. Nuclei were isolated from 50 to 100 mg of liver tissue. For this, samples were incubated with 500 μl of nuclei isolation buffer I (494 μl of nuclei EZ lysis buffer, 55 μl of 10× DNAseI buffer and 1 μl of DNAse I) for 15 min on ice. Then, 250 μl of nuclei EZ lysis buffer was added and samples were vortexed vigorously (4 cycles of 2 s “on” and 1 s “off”). After centrifuging the samples at 500 *g* for 5 min, the pellets were incubated with 250 μl of nuclei isolation buffer II (246.5 μl of nuclei EZ lysis buffer, 27.5 μl of 10× DNAseI buffer and 1 μl of DNAse I) for 10 min on ice. Then, 500 μl of nuclei EZ lysis buffer was added and samples were vortexed vigorously as described above. After centrifuging the samples at 500 *g* for 5 min, the pellets were incubated in 500 μl of nuclei EZ lysis buffer for 20 min on ice. Residual debris was removed by filtration through a 40‐μm cell strainer followed by centrifugation at 500 *g* for 5 min. After washing the nuclei pellet with 1× PBS, nuclei were counted using a hemocytometer. 100,000 nuclei were then used for ATAC‐seq library preparation following the Omni‐ATAC protocol. Libraries were sequenced in paired‐end mode on an Illumina HiSeq 2500 platform.

#### ChIP‐seq library preparation

ChIP‐seq libraries were prepared from two independent biological replicates per age group (young and aged). Unless otherwise stated, the entire procedure was performed at 4°C or on ice. Freshly harvested liver tissue was washed four times with ice‐cold 1× PBS (Gibco), cut on ice into small pieces and washed three more times with 1× PBS. The tissue was then cross‐linked with 1% formaldehyde and homogenized in a pre‐chilled Dounce tissue homogenizer using a loose pestle (15 strokes). After incubation for 10 min rocking at room temperature, the cross‐linking reaction was quenched with the addition of glycine to a final concentration of 0.125 M. After incubation for 5 min rocking at room temperature, the samples were centrifuged at 3,260 *g* for 5 min and the supernatant was discarded. 300 mg of cross‐linked tissue was lysed in 2 ml lysis buffer (50 mM Hepes pH 7.9, 140 mM NaCl, 1 mM EDTA, 10% glycerol, 0.5% NP‐40, 0.25% Triton x‐100, 0.5 μg/ml leupeptin, 0.7 μg/ml pepstatin A, 0.5 mM PMSF) and homogenized in a pre‐chilled Dounce tissue homogenizer using both a loose and tight pestle (15 strokes each). After the addition of 10 ml of lysis buffer, the samples were incubated on ice for 20 min and centrifuged at 3,260 *g* for 5 min. Nuclei pellets were washed by resuspending twice in 10 ml wash buffer (10 mM Tris pH 8.1, 200 mM NaCl, 1 mM EDTA, 0.5 mM EGTA, 0.5 μg/ml leupeptin, 0.7 μg/ml pepstatin A, 0.5 mM PMSF, 5 mM sodium butyrate) and centrifuging at 3,260 *g* for 5 min and then washing with 4 ml shearing buffer (0.1% SDS, 1 mM EDTA, 10 mM Tris pH 8.0, 0.5 μg/ml leupeptin, 0.7 μg/ml pepstatin A, 0.5 mM PMSF, 5 mM sodium butyrate) without disturbing the pellet. Then, pellets were resuspended in 2 ml shearing buffer and sonicated using a Focused Ultrasonicator M220 (Covaris). Sonication was performed in two rounds using two different sonication conditions: mild (peak power: 75, duty factor: 10.0, cycles per burst: 200, average power: 10.0, temperature range: 5–7°C) and intense (peak power: 75, duty factor: 25.4, cycles per burst: 200, average power: 19.1, temperature range: 5–7°C). Between sonication rounds, samples were centrifuged at 1,500 *g* for 5 min. After sonication, cellular debris was precipitated by centrifugation at 14,000 *g* for 20 min. An aliquot of clear supernatant was taken as ChIP input control (10 μg of chromatin). For chromatin immunoprecipitation, 25 μg of DNA was combined with 1% Triton X‐100, 150 mM NaCl, and SPT4 antibody (1:100 dilution, Cell Signaling Technology, catalog number: 64828, lot number: 1). Samples were incubated rotating at 4°C overnight. Magnetic protein G Dynabeads (Invitrogen) were equilibrated by washing three times with IP buffer (1% Triton, 0.1% SDS, 1 mM EDTA, 10 mM Tris pH 8.0, 150 mM NaCl). The immunoprecipitation reactions were then incubated with the beads at 4°C for 90 min. The beads were subsequently washed twice with each TSE‐150 (1% Triton, 0.1% SDS, 2 mM EDTA, 20 mM Tris pH 8.0, 150 mM NaCl) and TSE‐500 buffer (1% Triton, 0.1% SDS, 2 mM EDTA, 20 mM Tris 8.0, 500 mM NaCl) and once with each LiCl (0.25 M LiCl, 1% NP‐40, 1% sodium deoxycholate, 1 mM EDTA, 10 mM Tris pH 8.0) and TE buffer (1 mM EDTA, 10 mM Tris pH 8.0). The beads were then incubated in 45 μl PK digestion buffer (20 mM Hepes pH 7.5, 1 mM EDTA pH 8.0, 0.5% SDS) supplemented with 3 μl RNAse A (1 mg/ml stock, DNAse free, Thermo Fischer Scientific) at 37°C for 30 min. After the addition of 5 μl proteinase K (1 mg/ml stock, Thermo Fischer Scientific), samples were incubated at 50°C for 30 min with periodic vortexing. Reverse cross‐linking for both input control and ChIP samples was performed by adding NaCl to a final concentration of 0.3 M to the supernatant and incubating at 65°C overnight. DNA was purified using the Nucleospin Gel and PCR Clean‐up kit (Macherey‐Nagel) by following the manufacturer's instructions with slight modifications. After adding 5 volumes of buffer NTB, samples were centrifuged at 11,000 *g* for 30 s and washed twice with 650 μl NT3 buffer. Buffer remnants were removed by centrifuging at 11,000 *g* for 1 min. DNA was eluted in adding 45 μl RNase‐free water. Library preparation was performed as previously described (Tessarz *et al*, 2014). Libraries were sequenced on an Illumina HiSeq 2500 platform (paired‐end, 50‐bp reads).

#### CUT&RUN

For the CUT&RUN, 50 mg of four young and four old mouse liver tissues was used. Five million nuclei were extracted using the same process described for the ATAC‐seq experiment. CUT&RUN was performed on 1 million nuclei per sample as previously described with modifications (Skene & Henikoff, 2017). Nuclei were prepared using the Nuclei EZ prep kit (Sigma‐Aldrich, NUC‐101). Subsequently, nuclei were washed twice with Wash Buffer (20 mM HEPES, 150 mM NaCl, 0.5 mM Spermidine) and bound to activated Concanavalin A beads for 10 min at room temperature. Cell‐bead suspension was then resuspended in antibody buffer (20 mM MHEPES, 150 mM NaCl, 0.5 mM Spermidine, 0.05% Digitonin, 2 mM EDTA) and incubated overnight at 4°C with S2‐phospho (ab5095), S5‐phospho (ab 5,408) and PolII‐CTD (ab26721) antibodies from Abcam, in a concentration 1:250. Cell‐bead suspension was then washed twice with Digitonin Buffer (20 mM HEPES, 150 mM NaCl, 0.5 mM Spermidine, 0.05% Digitonin) and incubated with 2.5 μl EpiCypher CUTANA pAG‐MNase, for 10 min. After washing samples twice with Digitonin buffer, 1 μl 100 mM CaCl2 was added to samples which were incubated for 2 h at 4°C, rotating. To stop the reaction, STOP buffer (340 mM Nacl, 20 mm EDTA, 4 mM EGTA, 0.02%Digitonin, 50 μg/ml RNase A, 50 μg/ml Glycogen, sonicated yeast DNA as spike‐in) was added in each tube. Samples were incubated at 37°C for 10 min at 500 rpm, and after centrifugation, liquid was collected, DNA was purified using Zymo DNA clean concentrator kit. Libraries were prepared for sequencing as previously described (Tessarz *et al*, 2014) with 12 cycles of PCR.

#### RNA‐seq

Total RNA was isolated from four young and four old murine livers using the RNA extraction kit (Direct‐zol RNA MiniPrep, Zymoresearch), following the manufacturer's protocol. RNA libraries were created using the NEBNext Ultra II Directional RNA Library Prep Kit for Illumina at the MPI for Plant Breeding. Libraries were sequenced as single‐end 150 bp reads on Illumina HiSeq 4000.

#### Cellular fractionation

Cytoplasmic, soluble nuclear, and chromatin‐bound protein extracts were prepared by stepwise separation using Subcellular Protein Fractionation Kit for Tissues (Thermo Fisher Scientific, 87790). For this purpose, whole livers were harvested, snap‐frozen in liquid nitrogen, and stored at −80°C. Liver tissue was homogenized in liquid nitrogen using mortar and pestle and by avoiding thawing of the tissue at any time. 200 mg of each homogenized liver sample was resuspended in 2 ml of ice‐cold Cytoplasmic Extraction Buffer (CEB) supplemented with Halt Protease Inhibitor Cocktail (Thermo Fisher Scientific, 87786 at 1:100) and 1 mM sodium metavanadate (NaVO_3_) and processed as outlined by the manufacturer's protocol. Fractions were run on western blots and incubated with antibodies against Pol II (Abcam, ab817), Pol II Ser2 phospho (Abcam, ab5095), and Pol II Ser 5 phospho (Abcam, ab5408).

#### Mass spectrometry‐based proteomics

For proteomic analysis of whole tissue extract, a small fraction of pulverized liver tissue (approximately one full laboratory spatula with a 4‐mm diameter) from four young and five aged biological replicates was used. Samples were incubated in 100 μl of freshly prepared lysis buffer (6 M Guanidinium chloride [GuCl], 2.5 mM tris(2‐carboxyethyl) phosphine [TCEP], 10 mM CAA, 100 mM Tris–HCl) at 95°C for 10 min and then sonicated using a Bioruptor 300 (Diagenode) (20 cycles of 30 s on, 30 s off). Sonicated samples were centrifuged for 20 min at 20,000 *g*, and the protein concentration in the supernatant was measured using a spectrophotometer (NanoDrop 2000, Thermo Scientific). 300 μg of proteins were diluted 10 times with 20 mM Tris, before adding trypsin (1 μg/μl, Promega, Mass Spectrometry grade) to a final concentration of 1:200 (w/w). Digestion reactions were incubated at 37°C overnight. Peptides were acidified in 100 μl of 0.1% formic acid and then desalted using StageTips (Empore), which were equilibrated by centrifuging with 200 μl of 0.1% formic acid for 90 s in a StageTip centrifuge (Empore). After centrifuging the peptides through the columns for 2 min, columns were washed twice using 200 μl of 0.1% formic acid. Peptides were eluted in 100 μl of 40% acetonitrile/0.1% formic acid by centrifuging at 300 *g* for 4 min. Peptides were then dried in a Speed‐Vac (Eppendorf) at 45°C for 45 min and resuspended in 20 μl of 0.1% formic acid. 4 μg of peptides was dried in the Speed‐Vac for another 15 min at 45°C, was dried out, and reconstituted in 9 μl of 0.1 M TEAB. Tandem mass tag (TMTpro, Thermo Fisher Scientific cat. No A44522) labeling was carried out according to the manufacturer's instruction with the following changes: 0.5 mg of TMTPro reagent was resuspended with 33 μl of anhydrous ACN. Seven microliters of TMTPro reagent in ACN was added to 9 μl of clean peptide in 0.1 M TEAB. The final ACN concentration was 43.75%, and the ratio of peptides to TMTPro reagent was 1:20. After 60 min of incubation, the reaction was quenched with 2 μl of 5% hydroxylamine. Labeled peptides were pooled, dried, resuspended in 200 μl of 0.1% formic acid (FA), split into two equal parts, and desalted using homemade STAGE tips (Li *et al*, 2021). One of the two parts was fractionated on a 1 mm × 150 mm ACQUITY column, packed with 130 Å, 1.7 μm C18 particles (Waters cat. no SKU: 186006935), using an Ultimate 3000 UHPLC (Thermo Fisher Scientific). Peptides were separated at a flow of 30 μl/min with a 88 min segmented gradient from 1% to 50% buffer B for 85 min and from 50% to 95% buffer B for 3 min; buffer A was 5% ACN, 10 mM ammonium bicarbonate (ABC), and buffer B was 80% ACN, 10 mM ABC. Fractions were collected every three minutes, and fractions were pooled in two passes (1 + 17, 2 + 18 … etc.) and dried in a vacuum centrifuge (Eppendorf). Dried fractions were resuspended in 0.1% formic acid (FA) and separated on a 50 cm, 75 μm Acclaim PepMap column (Thermo Fisher Scientific, Product No. 164942) and analyzed on an Orbitrap Lumos Tribrid mass spectrometer (Thermo Fisher Scientific) equipped with a FAIMS device (Thermo Fisher Scientific). The FAIMS device was operated in two compensation voltages, −50 and −70 V. Synchronous precursor selection based MS3 was used for the acquisition of the TMTPro reporter ion signals. Peptide separations were performed on an EASY‐nLC1200 using a 90 min linear gradient from 6% to 31% buffer; buffer A was 0.1% FA, and buffer B was 0.1% FA, 80% ACN. The analytical column was operated at 50°C. Raw files were split based on the FAIMS compensation voltage using FreeStyle (Thermo Fisher Scientific). Proteomics data were analyzed using MaxQuant, version 1.6.17.0 (Cox & Mann, 2008). The isotope purity correction factors, provided by the manufacturer, were included in the analysis. Differential expression analysis was performed using limma, version 3.34.9 (Ritchie *et al*, 2015) in R, version 3.4.3 (Table EV6).

#### Tissue NET‐seq (tNET‐seq)

We modified the original NET‐seq protocol (Mayer & Churchman, 2016) for application in murine liver tissue and named this new adaptation of the protocol tissue NET‐seq (tNET‐seq). The main change in the protocol pertains to the nuclei isolation. Fresh liver tissue was placed in ice‐cold PBS, cut into smaller pieces, and homogenized in 3 ml of nuclei isolation buffer (nuclei EZ lysis buffer [Sigma, NUC‐101], 1× Halt™ protease inhibitor cocktail [Thermo Fisher, 87786], 40 U RNasin [Promega, N2511], 25 μM α‐amanitin [Sigma, A2263]) using a Dounce tissue homogenizer. In total, whole livers from nine mice (3 per age group: young, middle‐aged, and aged) were processed immediately after sacrifice. The livers from young mice weighed between 1 and 1.5 g, while those from middle‐aged and aged mice were heavier with 2–2.5 g. The entire procedure was performed on ice or at 4°C using RNase‐free equipment. After complete homogenization, samples were transferred to a 15 ml tube. 3 ml of nuclei isolation buffer was added to the remaining pieces in the tissue homogenizer, homogenized further, and transferred to the same 15 ml tube. Following an incubation on ice for 5 min, samples were passed through a 70‐μm cell strainer and nuclei were collected by centrifuging at 500 *g* for 20 min. The nuclei pellet was resuspended in 4 ml of nuclei isolation buffer and incubated on ice for 5 min. Nuclei were collected by centrifuging at 500 *g* for 5 min. To remove cytoplasmic remnants, the nuclei pellet was washed with 1,600 μl nuclei wash buffer (1× PBS, 0.1% [v/v] Triton X‐100, 1 mM EDTA, 25 μM α‐amanitin, 40 U RNasin, 1× Halt™ protease inhibitor cocktail) and centrifuged at 1,150 *g* for 5 min. Washing was repeated with 800 μl nuclei wash buffer. Then, the pellet was gently resuspended in 200 μl of glycerol buffer (20 mM Tris–HCl [pH 8.0], 75 mM NaCl, 0.5 mM EDTA, 50% [v/v] glycerol, 0.85 mM DTT, 25 μM α‐amanitin, 10 U RNasin, 1× Halt™ protease inhibitor cocktail) using a cut 1,000‐μl tip and transferred to a new 1.5‐ml RNase‐free microcentrifuge tube. Nuclei were lysed in 400 μl of nuclei lysis buffer (20 mM HEPES (pH 7.5), 300 mM NaCl, 1% (v/v) NP‐40, 1 M urea, 0.2 mM EDTA, 1 mM DTT, 25 μM α‐amanitin, 10 U RNasin, 1× Halt™ protease inhibitor cocktail) and mixed by pulsed vortexing, followed by incubation on ice for 20 min. After assessing nuclei lysis of a small aliquot under a light microscope, samples were centrifuged at 18,500 *g* for 2 min. The supernatant (nucleoplasmic fraction) was completely removed, and the chromatin pellet was resuspended in 50 μl chromatin resuspension solution (PBS, supplemented with 25 μM α‐amanitin, 20 U RNasin, 1× Halt™ protease inhibitor cocktail). A version of the protocol is maintained at protocols.io https://doi.org/10.17504/protocols.io.kxygxzj3dv8j/v1.

#### Computational analyses

##### Primary data processing

Primary sequencing data were quality controlled using FastQC (v. 0.11.5, http://www.bioinformatics.babraham.ac.uk/projects/fastqc). Single‐end tNET‐seq reads were trimmed to 50‐bp length using cutadapt (v. 1.13) (Martin, 2011) and processed using custom Python scripts (adapted from https://github.com/BradnerLab/netseq). Paired‐end ATAC‐seq reads were trimmed to remove Tn5 transposase adapter sequence using cutadapt (v. 1.13) (Martin, 2011) with the parameter –minimum‐length = 20. Reads were then aligned to the GRCm38 reference genome (Ensembl release 99) using Bowtie2 (v. 2.4.1) (Langmead & Salzberg, 2012) by enabling soft clipping (‐‐local) and the alignment of fragments up to 2 kb (−X 2000). Aligned reads were then filtered for high‐quality (MAPQ > 10) and properly paired (samtools flag 0x2) (Li *et al*, 2009) reads. Finally, reads arising from PCR duplicates and those aligned to the mitochondrial genome were removed using PicardTools (v. 2.21.4, http://broadinstitute.github.io/picard/) and samtools (v. 1.10) in combination with grep, respectively.

ChIP‐seq reads were aligned to the GRCm38 reference genome (Ensembl release 99) using Bowtie2 (v. 2.4.1) by enabling soft clipping (‐‐local). Aligned reads were then filtered for high quality (MAPQ > 10). Reads arising from PCR duplicates and those aligned to the mitochondrial genome were removed using PicardTools (v. 2.21.4) and samtools (v. 1.10) in combination with grep, respectively.

The quality of aligned reads was assessed using Rsamtools (v. 2.2.3) (Morgan, 2013), Additionally, fragment size distribution of ATAC‐seq data was assessed using ATACseqQC (v. 1.14.4) (Ou *et al*, 2018).

##### Peak calling and annotation

ATAC‐seq and ChIP‐seq peaks were called on aligned and filtered BAM files using MACS2 (v. 2.2.7; preprint: Gaspar, 2018). Where applicable, peak calling was performed in paired‐end mode (−f BAMPE). For TF and histone ChIP‐seq, the corresponding input libraries and total H3 ChIP‐seq samples were used to determine the local background levels, respectively. Peaks displaying an FDR < 0.05 were considered as statistically significant.

The fraction of reads in peaks (FRiP) and fraction of reads in blacklisted regions (FRiBL) was determined using the R package ChIPQC (v. 1.21.0) (Carroll & Stark, 2017). Peaks falling in ENCODE blacklist regions were subsequently removed. Peaks were annotated using the R packages ChIPseeker (v. 1.26.2) (Yu *et al*, 2015) and ChIPpeakAnno (v. 3.24.2) (Zhu *et al*, 2010). The promoter region was consistently defined as TSS ± 200 bp.

##### CUT&RUN analysis

The fastq reads were mapped to the mm10 genome using bowtie2 (V. 2.3.5) (Langmead & Salzberg, 2012). Duplicates were removed using MarkDuplicates program of Picard Tools (V. 2.21.4) (http://broadinstitute.github.io/picard). For mapping spike‐in fragments to the yeast genome, the “–no‐overlap–no‐dovetail” options were set and mapped to a repeat‐masked version of the yeast genome (R64) to avoid crossmapping of the mouse genome to that of the yeast genome. Peak calibration was done as described (Skene & Henikoff, 2017). The peaks were then called using the SEACR package (V. 1.3) in “stringent”‐mode (Meers *et al*, 2019). For visualization purposes, the replicates were merged using “samtools merge” (V. 1.9), and the bigwig files were generated using “bamCoverage –normalizeUsing RPGC.”

##### MNase‐seq

Mnase‐seq data were downloaded from GSE58005 (Bochkis *et al*, 2014). Analysis of the MNase samples was performed using the nf‐core framework (Ewels *et al*, 2020) using the mnase‐seq module. Coverage plot was created using the ngsplot (Shen *et al*, 2014).

##### Gene selection

Annotation files for GRCm38 were retrieved from Ensembl (release 99). The genes included in the analysis were carefully selected to avoid contamination from transcription arising from other transcription units. Hence, only protein‐coding genes were considered that are longer than 2 kb and non‐overlapping within a region of 2.5 kb upstream of the TSS (start of the open reading frame of the canonical transcript) and downstream of the TES (end of the open reading frame of the canonical transcript ± 200 bp) (*n* = 12,460). In case of multiple transcript isoforms, the canonical transcript is defined as the longest coding sequence, if the gene has translated transcripts, or as the longest cDNA. To ensure that only genes with sufficient coverage were included, the list was further filtered to contain only genes with RPKM > 1 (considering only uniquely aligned and filtered reads) (*n* = 3,280).

##### Differential accessibility analysis with ATAC‐seq

Overlapping peaks that were called in different ATAC‐seq samples were resolved by defining a consensus peak set containing non‐redundant peaks present in at least two biological replicates regardless of condition (i.e., age). Reads overlapping consensus peak regions were recorded using featureCounts from the R package Rsubread (v. 2.0.1) (Liao *et al*, 2019). Differential accessibility analysis was performed using the R package DESeq2 (v. 1.26.0) (Love *et al*, 2014). Regions with an FDR < 5% were considered to be statistically significant.

##### Differential expression analysis with RNA‐seq

Differential expression analysis was performed using DESeq2 (v. 1.26.0) (Love *et al*, 2014). Genes with an FDR < 5% were considered to be statistically significant.

##### Recording Pol II density at nucleotide resolution

Pol II density was calculated by recording the genomic position of the 5′ end of each tNET‐seq read, which corresponds to the 3′ end of the original nascent RNA and represents the exact genomic position of Pol II. For this, bedtools (v. 2.29.2) (Quinlan & Hall, 2010) genomecov with the parameters ‐dz and ‐5 was used.

##### Differential transcription analysis with tNET‐seq

Pol II density in gene bodies of tNET genes was retrieved using bedtools (v. 2.29.2) (Quinlan & Hall, 2010) intersect and the read count matrix of genome‐wide Pol II densities. For this, the gene body was defined as 200 bp downstream of the TSS to 200 bp upstream of the TES. To identify differentially transcribed genes, differential analysis of Pol II density in gene bodies was performed using the R package DESeq2 (v. 1.26.0) (Love *et al*, 2014). Here, only sense transcription was considered (i.e., tNET‐seq reads sharing the same orientation as the annotation). Genes with an FDR < 5% were considered to be statistically significant.

##### Trajectory analysis of nascent transcription with tNET‐seq

To estimate nascent transcription trajectories during aging, we performed a likelihood ratio test using the R package DESeq2 (v. 1.26.0) (Love *et al*, 2014). Regions with an FDR < 5% were considered to be statistically significant. Then, we identified common patterns using the R package DEGreport (v. 1.26.0) (Pantano, 2019). In brief, rlog‐transformed counts of significantly differentially transcribed genes were used for calculating pair‐wise gene expression among conditions using Kendall's rank. Divisive hierarchical clustering (DIANA) was then used on the gene–gene distance matrix for identifying groups of genes with similar trajectories. *Z*‐scores of these genes were visualized.

##### Pol II pausing index

To quantify promoter‐proximal pausing, we calculated the Pol II pausing index, which is defined as the ratio between the average Pol II density in the promoter‐proximal region (defined here as TSS ± 200 bp) over that in the gene body (defined here as TSS +200 bp to TES −200 bp).

For computing the Pol II pausing index, RPM‐normalized Pol II coverage files were generated using bedtools (v. 2.29.2) (Quinlan & Hall, 2010) genomecov with the parameters ‐dz, ‐5 and ‐scale 1/number of aligned reads in mio. The number of aligned reads after pre‐processing was obtained using samtools view (v 1.10) (Li *et al*, 2009) with the flag ‐c and ‐F 260 to output the number of primary aligned reads only.

The RPM‐normalized count matrices were intersected with annotation files for both promoter‐proximal and gene body regions using bedtools (v. 2.29.2) (Quinlan & Hall, 2010) intersect. After normalizing for region length, the pausing index was calculated by dividing the mean normalized Pol II density in the promoter‐proximal region by the mean normalized Pol II density in the gene body region for each tNET gene. Here, only sense transcription was considered (i.e., tNET‐seq reads sharing the same orientation as the annotation). Extreme data points were removed from the pausing analysis by retaining only genes with a pausing index ≤10 in all samples (*n* = 109 genes removed).

##### Pol II pausing index and promoter accessibility

To allow for a direct comparison between promoter accessibility and the Pol II pausing index, the ATAC‐seq data were processed analogous to the tNET‐seq data. In brief, RPM‐normalized chromatin accessibility was calculated by recording the genomic position of the 5′ end of each ATAC‐seq read using bedtools (v. 2.29.2) (Quinlan & Hall, 2010) genomecov as described above. We then computed chromatin accessibility in promoter‐proximal regions (TSS ±200 bp) using bedtools (v. 2.29.2) intersect. After normalizing for region length, the log_2_‐fold change in promoter accessibility in aged versus young mice was compared with the log_2_‐fold change in pausing index in aged versus young mice.

##### Identification of active enhancers in murine liver tissue

For identifying active enhancers in murine liver tissue, we combined our accessibility data (ATAC‐seq) with publicly available histone modification data (H3K27ac and H3K4me3 ChIP‐seq). For each dataset, we defined a consensus peak set containing peaks present in all samples regardless of biological condition (i.e., age). Enhancers were then identified as H3K27ac peaks that (i) do not overlap H3K4me3 peaks, (ii) do not fall within TSS ± 1 kb, and (iii) overlap accessible sites identified through ATAC‐seq. This resulted in the identification of 8,855 enhancer regions active in murine liver tissue.

##### Functional enrichment analysis

GO databases were queried using the R package clusterProfiler (v. 3.14.3) (Yu *et al*, 2012). To test for over‐representation, the complete gene list of the mouse database (Mm.eg.db, v. 3.12.0) served as background. After removing semantic redundancy, GO terms were ranked by adjusted *P*‐value (Benjamini‐Hochberg procedure, FDR < 0.05).

##### Visualization

For visualization purposes, aligned and filtered BAM files were converted to bigwig coverage tracks using Deeptools (v. 3.5.1) (Ramírez *et al*, 2014). For ATAC‐seq and tNET‐seq, a bin size of 1 bp was used, while ChIP‐seq reads were extended and counted in 10‐bp bins. Bigwig files were normalized to 1× coverage (‐‐normalizeUsing RPGC). For tNET‐seq, only the position of the 5′ end of the sequencing read was recorded (‐‐Offset 1).

Metagene profiles and heatmaps of mean signal enrichment were generated using Deeptools (v 3.5.1) and files normalized to 1× coverage as input. For tNET‐seq, reads sharing the same or opposite orientation with the annotation were assigned as sense or antisense, respectively. Biological replicates were visualized either separately or merged prior to visualization.

Sample‐by‐sample correlation and principal component analyses were performed using rlog‐transformed read counts via DESeq2 (v. 1.26.0) (Love *et al*, 2014). Single‐gene sequencing tracks were visualized with Integrative Genomics Viewer (v. 2.8.0) (Robinson *et al*, 2011).

##### Reproducibility and statistical analysis

R (v. 3.6.3) and Python (v. 3.9.0) were used for all analyses. Statistical parameters and significance are reported in the figures and figure legends. Whenever possible, we used non‐parametric statistical tests to avoid assuming the normality of data distributions. In cases of *P* < 2.2e‐16, please note that this is the notation used by the programing language R for reporting very small numbers, as 2.2e‐16 is the smallest number larger than 0 that can be stored by the floating system due to computer precision limits.

##### Publicly available analyzed datasets

Tabula Muris senis bulk RNA‐seq data (Schaum *et al*, 2020) was retrieved from Figshare as raw count table (https://figshare.com/projects/The_murine_transcriptome_reveals_global_aging_nodes_with_organ‐specific_phase_and_amplitude/65126).

Additionally, we analyzed publicly available histone ChIP‐seq data for H3, H3K27me3 and H3K4me3. Raw data originating liver tissue of 3‐, 12‐, and 29‐month‐old male mice were retrieved from the NCBI BioProject database (PRJNA281127, https://www.omicsdi.org/dataset/omics_ena_project/PRJNA281127) (Benayoun *et al*, 2019). Liver MNase data were downloaded from GSE58005 (https://www.ncbi.nlm.nih.gov/geo/query/acc.cgi?acc=GSE58005) (Bochkis *et al*, 2014).

## Author contributions

**Mihaela Bozukova:** Conceptualization; data curation; formal analysis; investigation; methodology; writing – original draft. **Chrysa Nikopoulou:** Investigation; writing – review and editing. **Niklas Kleinenkuhnen:** Formal analysis; writing – review and editing. **Dora Grbavac:** Investigation; writing – review and editing. **Katrin Goetsch:** Investigation; writing – review and editing. **Peter Tessarz:** Conceptualization; funding acquisition; writing – original draft; project administration; writing – review and editing.

## Disclosure and competing interests statement

The authors declare that they have no conflict of interest.

## Supporting information




Appendix

Expanded View Figures PDF

Table EV1

Table EV2

Table EV3

Table EV4

Table EV5

Table EV6

## Data Availability

All sequencing data generated in this study (ATAC‐, NET‐, RNA‐, and ChIP‐seq as well as CUT&RUN) are available at Gene Expression Omnibus, accession number: GSE198189 (https://www.ncbi.nlm.nih.gov/geo/query/acc.cgi?acc=GSE198189). The mass spectrometry proteomics data have been deposited to the ProteomeXchange Consortium via the PRIDE (Perez‐Riverol *et al*, 2019) partner repository with the dataset identifier PXD034564 (http://www.ebi.ac.uk/pride/archive/projects/PXD034564).
