# Supplementary material for: Aging is associated with increased chromatin accessibility and reduced polymerase pausing in liver
Source: Mol Syst Biol. 2022 Sep 9;18(9):e11002. doi: 10.15252/msb.202211002 (PMC9459415; doi:10.15252/msb.202211002)
Supplement: Supplementary file 1 — Appendix [file MSB-18-e11002-s008.pdf]

Appendix

Appendix Figures S1-5 (pp.1-5)

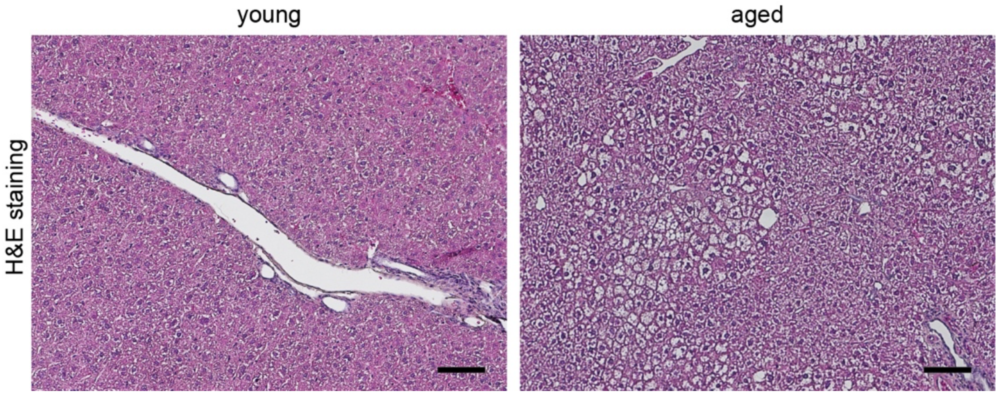

Appendix Figure S1: H&E staining of young and old liver. Representative images from H&E stainings on liver sections from a young and an old mouse. Scale bar=100μm.

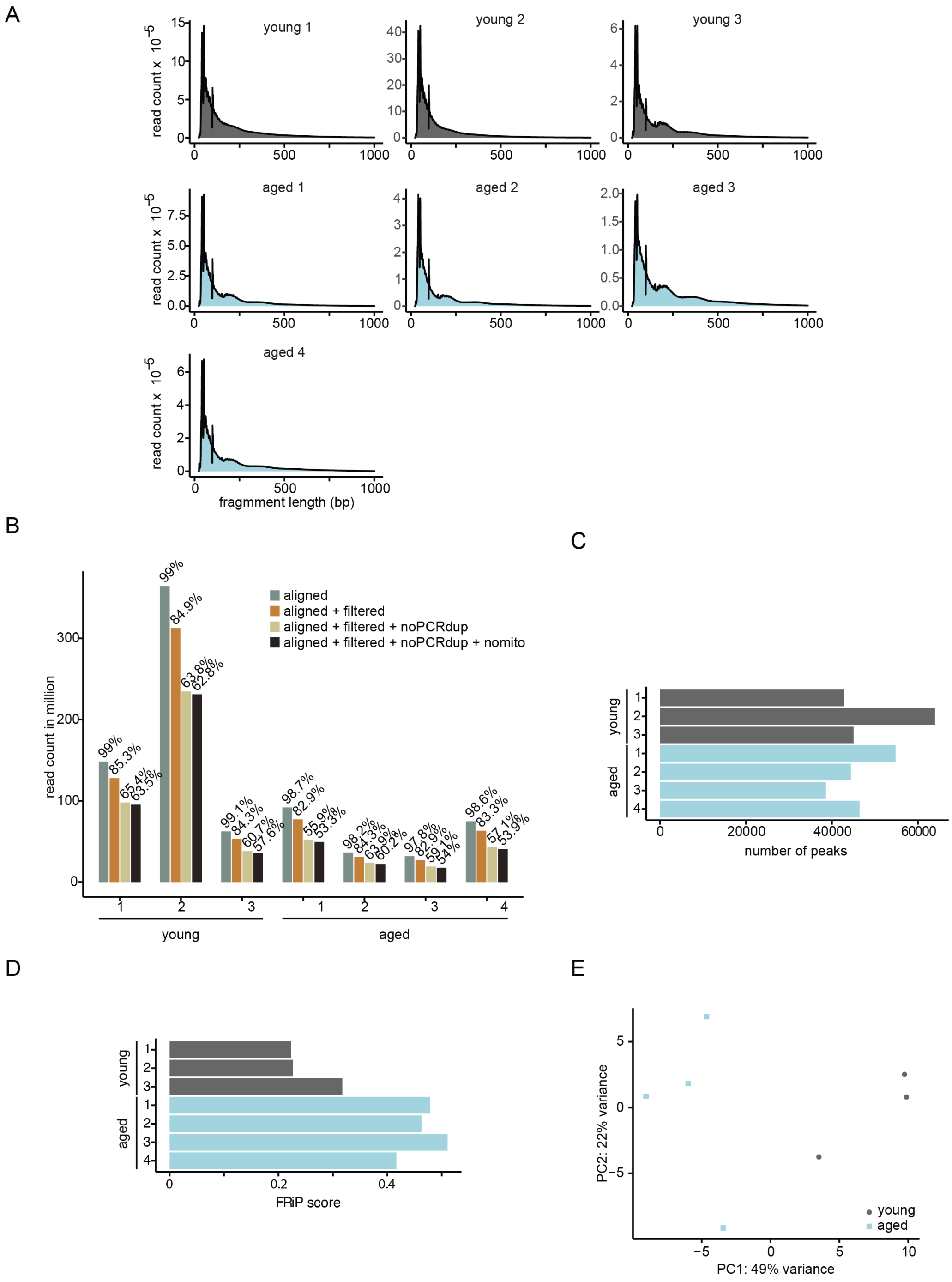

Appendix Figure S2: Quality control of ATAC-seq. A) Fragment size distribution of reads passing filtering criteria for each ATAC-seq library. Each curve represents one biological replicate (n = 3 and 4 young and aged mice, respectively). **B)** Number of ATAC-seq reads successfully aligned and passing filtering criteria. Aligned reads were filtered for high quality (MAPQ > 10) and PCR duplicates and mitochondrial reads were removed. **C)** Number of peak regions identified in each ATAC-seq library. **D)** Signal-to-noise ratio assessed by the fraction of reads in peaks (FRiP). **E)** Principal component analysis of chromatin accessibility profiles. Rlog-normalized read counts (DESeq2) in consensus peak regions identified by ATAC-seq are depicted. Percentage of variance accounted for by each principal component is indicated.

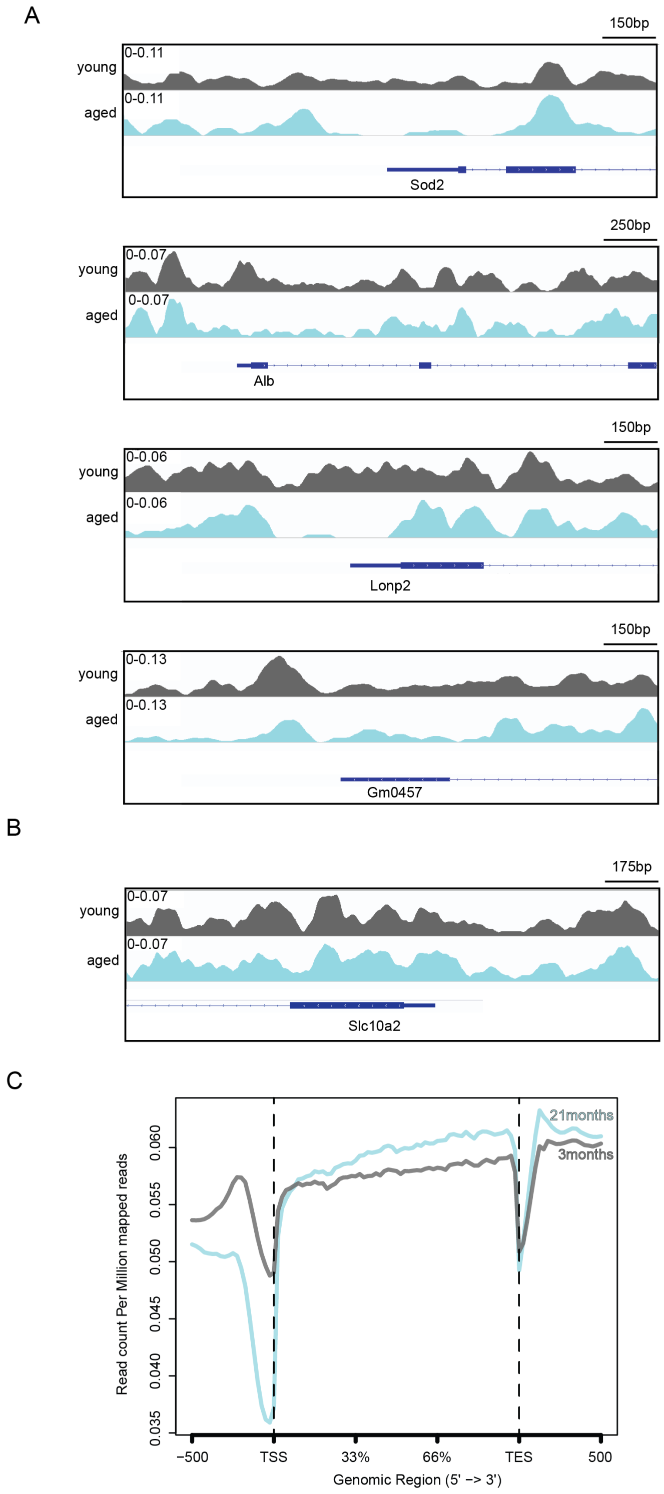

Appendix Figure S3: Accessibility of promoter regions as assessed by MNase-seq. Previously published MNase-seq data (Bochkis *et al.*, 2014) was used to corroborate the observed increase in promoter accessibility by ATAC-seq (see Fig. 1). A) Selected loci (see also Fig. 1F) that show an increase in promoter accessibility. B) Control locus that does not show a difference between young and aged liver. C) Metaprofile of MNase-seq data spanning a region from TSS – 500bp to TES +500bp.

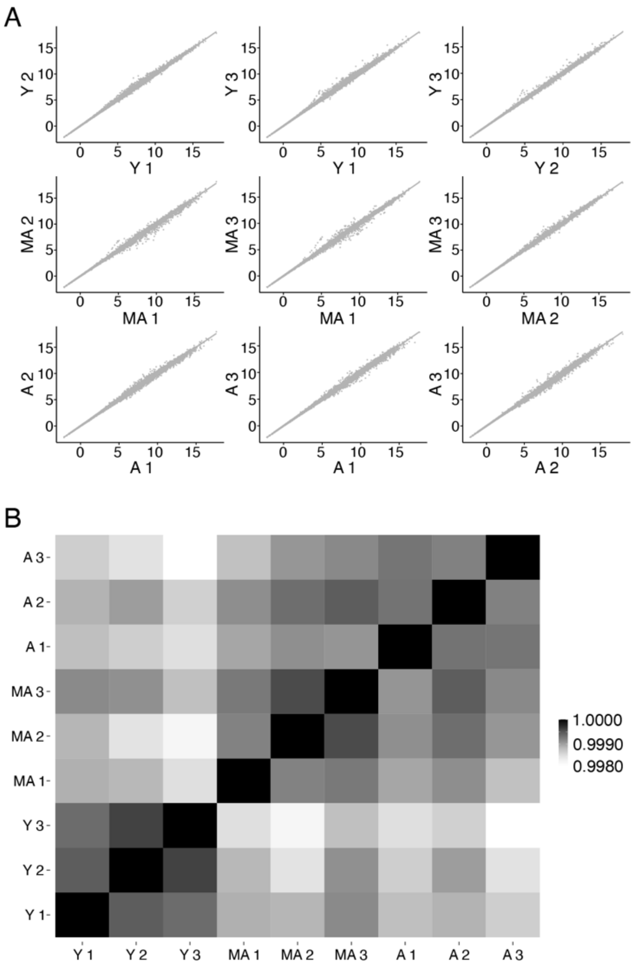

Appendix Figure S4: Correlation between (t)NET-seq experiments. A) Pairwise comparison of tNET-seq samples. Normalized read counts (rlog transformation, DESeq2) in non-overlapping, protein-coding genes above 2 kb in size are reported. **B)** Heatmap of replicate correlations using normalized read counts as in A. The color code represents Pearson correlation coefficient.

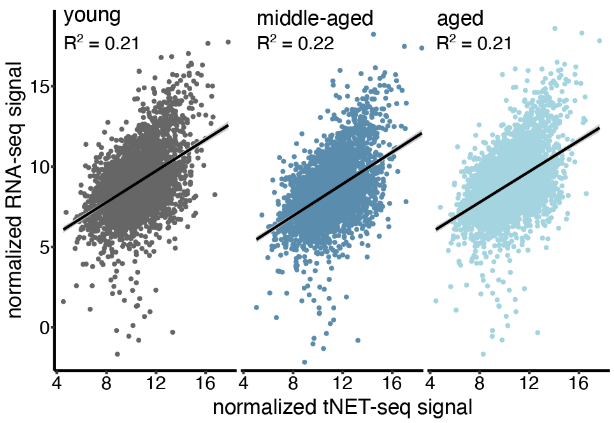

Appendix Figure S5: RNA-/NET-seq correlation. Correlation between nascent (gene-body Pol II density, tNET-seq) and steady-state transcription (RNA-seq). Mean normalized read counts (rlog transformation, DESeq2) of merged biological replicates from young, middle-aged and aged animals are reported.
