# Supplementary material for: Aging is associated with increased chromatin accessibility and reduced polymerase pausing in liver
Source: Mol Syst Biol. 2022 Sep 9;18(9):e11002. doi: 10.15252/msb.202211002 (PMC9459415; doi:10.15252/msb.202211002)
Supplement: Supplementary file 2 — Expanded View Figures PDF [file MSB-18-e11002-s009.pdf]

Expanded View Figures

Figure EV1. Promoter accessibility and functional annotation.

A Distance of differentially accessible sites to the nearest annotated TSS. *P*-value was calculated using a two-sided Wilcoxon rank-sum test. $N = 2{,}760$ and 1,931 sites with increased and decreased accessibility with age, respectively. Box plots consist of the median (central line), the 25th and 75th percentiles (box) and the highest/lowest value within 1.5 * interquartile range of the box (whiskers).

B, C GO term enrichment analysis for genes with increased (B) and decreased (C) promoter accessibility in the liver of aged mice. Only genes with a differentially accessible TSS were included in the analysis ($n = 1{,}945$ genes in total; 1,704 with increased and 241 with decreased promoter accessibility). Only the top five enriched GO terms ranked by adjusted *P*-value (Benjamini-Hochberg [BH] procedure, FDR < 0.05) are displayed. BH-adjusted *P*-values are reported for each GO term inside the respective bar.

Data information: ATAC-seq: young, $n = 3$; old, $n = 4$; all biological replicates.

© 2022 The Authors

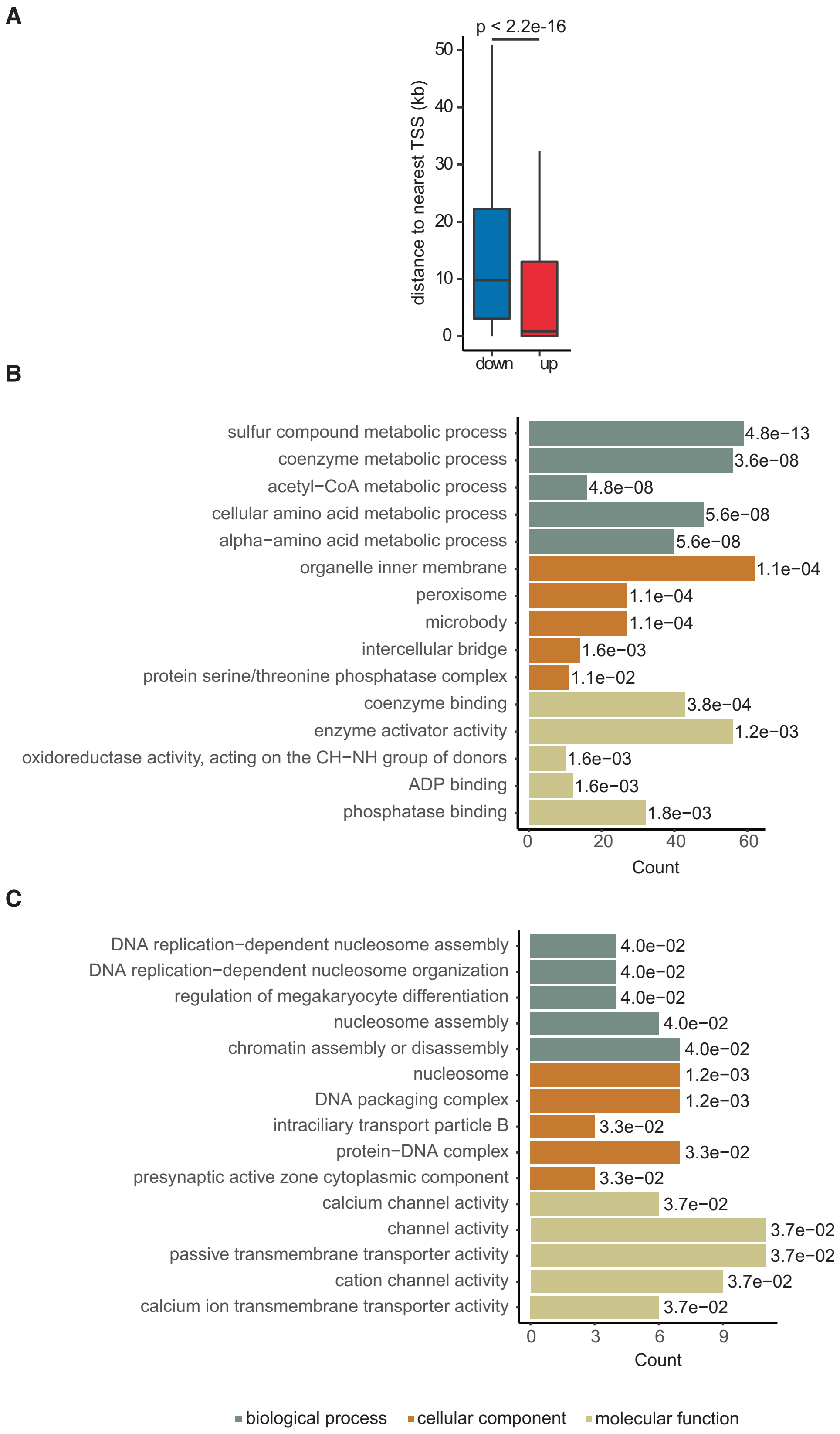
A
p < 2.2e-16
distance to nearest TSS (kb)
50
40
30
20
10
0
down
up
B
sulfur compound metabolic process
coenzyme metabolic process
acetyl−CoA metabolic process
cellular amino acid metabolic process
alpha−amino acid metabolic process
organelle inner membrane
peroxisome
microbody
intercellular bridge
protein serine/threonine phosphatase complex
coenzyme binding
enzyme activator activity
oxidoreductase activity, acting on the CH−NH group of donors
ADP binding
phosphatase binding
4.8e−13
3.6e−08
4.8e−08
5.6e−08
5.6e−08
1.1e−04
1.1e−04
1.1e−04
1.6e−03
1.1e−02
3.8e−04
1.2e−03
1.6e−03
1.6e−03
1.8e−03
0
20
40
60
Count
C
DNA replication−dependent nucleosome assembly
DNA replication−dependent nucleosome organization
regulation of megakaryocyte differentiation
nucleosome assembly
chromatin assembly or disassembly
nucleosome
DNA packaging complex
intraciliary transport particle B
protein−DNA complex
presynaptic active zone cytoplasmic component
calcium channel activity
channel activity
passive transmembrane transporter activity
cation channel activity
calcium ion transmembrane transporter activity
4.0e−02
4.0e−02
4.0e−02
4.0e−02
4.0e−02
1.2e−03
1.2e−03
3.3e−02
3.3e−02
3.3e−02
3.7e−02
3.7e−02
3.7e−02
3.7e−02
3.7e−02
0
3
6
9
Count
biological process
cellular component
molecular function

Figure EV1.

Figure EV2. Integration of RNA- and (t)NET-seq.

A PCA scatter plot of steady-state gene expression profiles assessed by RNA-seq. Percentage of variance accounted for by each principal component is indicated.

B Volcano plot of differentially expressed genes comparing middle-aged relative to young animals (FDR < 0.05, Wald test). 307 genes were up-regulated (red) and 194 down-regulated (blue).

C Volcano plot of differentially expressed genes in the liver of aged versus young animals (FDR < 0.05, Wald test). 127 genes were up-regulated (red) and 92 down-regulated (blue). $n = 3$–4 biological replicates per age group.

D Principal component analysis of nascent transcription assessed by tNET-seq. PCA was performed with the normalized read counts (rlog transformation, DESeq2) in gene bodies of non-overlapping, protein-coding genes above 2 kb in size. The percentage of variance accounted for by each principal component is indicated.

E Volcano plot of differentially transcribed genes (gene-body Pol II density) in middle-aged relative to young animals (FDR < 0.05, Wald test). 196 genes were up-regulated (red) and 171 down-regulated (blue).

F Volcano plot of differentially transcribed genes (gene-body Pol II density) in aged relative to young animals (FDR < 0.05, Wald test). 365 genes were up-regulated (red) and 352 down-regulated (blue). A, aged; MA, middle-aged; Y, young. $n = 3$ biological replicates per age group.

G Correlation between changes in nascent (gene-body Pol II density, tNET-seq) and steady-state transcription (RNA-seq, generated of mice from our standing aging cohort at the MPI for Biology of Ageing) of aged versus young animals.

H Violin and boxplots of changes in promoter accessibility (ATAC-seq), nascent transcription (gene-body Pol II density, tNET-seq) and steady-state transcription (RNA-seq, generated of mice from our standing aging cohort at the MPI for Biology of Ageing) in aged versus young mice. Only genes present in all three datasets are included here ($n = 2{,}698$ genes). Box plots consist of the median (central line), the 25th and 75th percentiles (box) and the highest/lowest value within 1.5 * interquartile range of the box (whiskers). Promoter defined as TSS ± 200 bp.

I Venn diagram of differentially transcribed (gene-body Pol II density, tNET-seq) and differentially expressed genes (RNA-seq, generated of mice from our standing aging cohort at the MPI for Biology of Ageing) in aged versus young mice. Only significantly changed genes (FDR < 0.05, Wald test) were included. Up- or down-regulated genes in both datasets are indicated in red and blue, respectively. Genes exhibiting divergent changes are indicated in grey.

Data information: ATAC-seq: young, $n = 3$; old, $n = 4$; all biological replicates; tNET-seq: three biological replicates per age group.

© 2022 The Authors

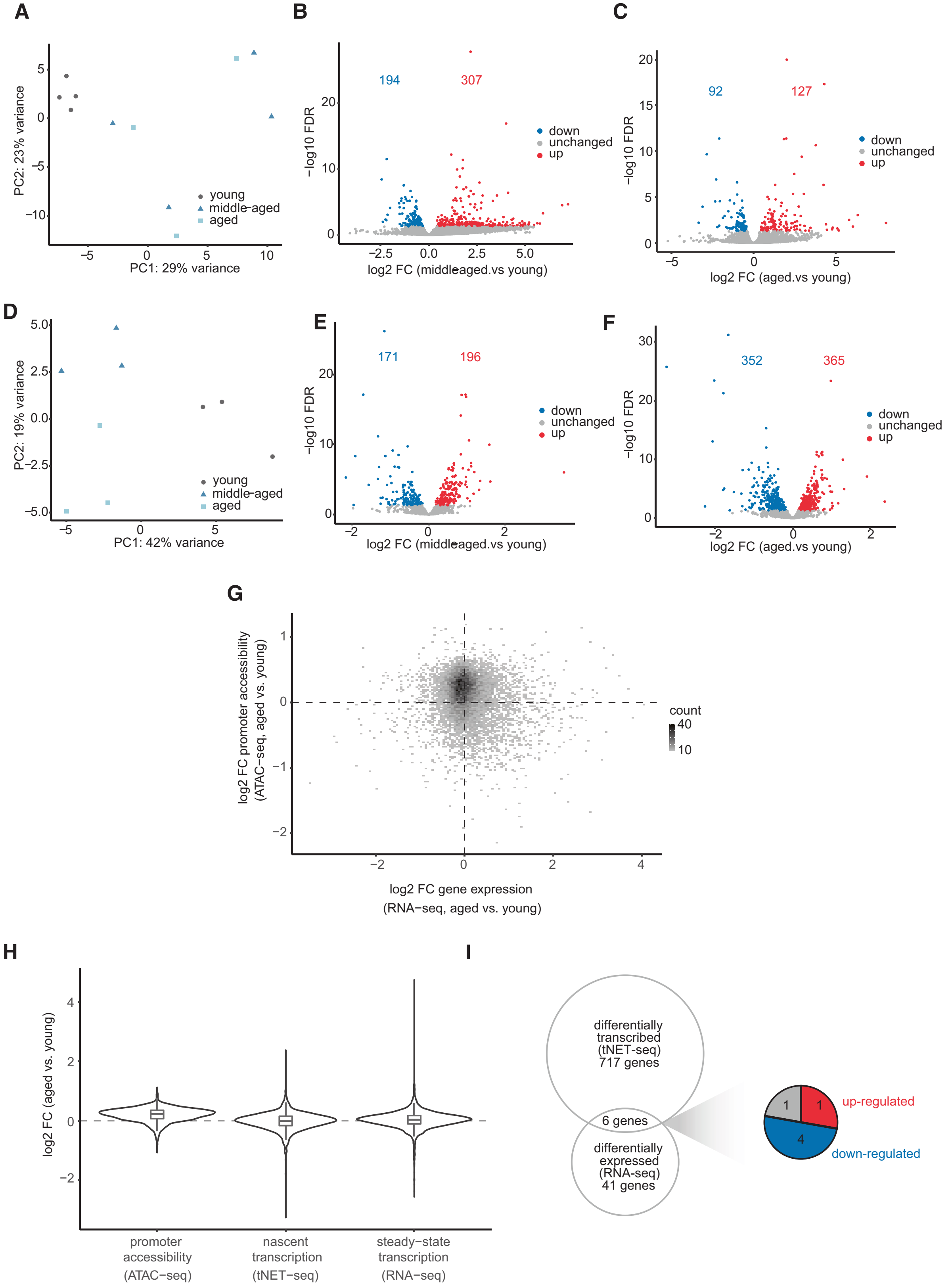
A
PC2: 23% variance
PC1: 29% variance
young
middle-aged
aged
B
194
307
−log10 FDR
log2 FC (middle-aged.vs young)
down
unchanged
up
C
92
127
−log10 FDR
log2 FC (aged.vs young)
down
unchanged
up
D
PC2: 19% variance
PC1: 42% variance
young
middle-aged
aged
E
171
196
−log10 FDR
log2 FC (middle-aged.vs young)
down
unchanged
up
F
352
365
−log10 FDR
log2 FC (aged.vs young)
down
unchanged
up
G
log2 FC promoter accessibility
(ATAC−seq, aged vs. young)
log2 FC gene expression
(RNA−seq, aged vs. young)
count
40
10
H
log2 FC (aged vs. young)
promoter accessibility (ATAC−seq)
nascent transcription (tNET−seq)
steady−state transcription (RNA−seq)
I
differentially transcribed (tNET−seq) 717 genes
6 genes
differentially expressed (RNA-seq) 41 genes
up-regulated
down-regulated
1
1
4

Figure EV2.

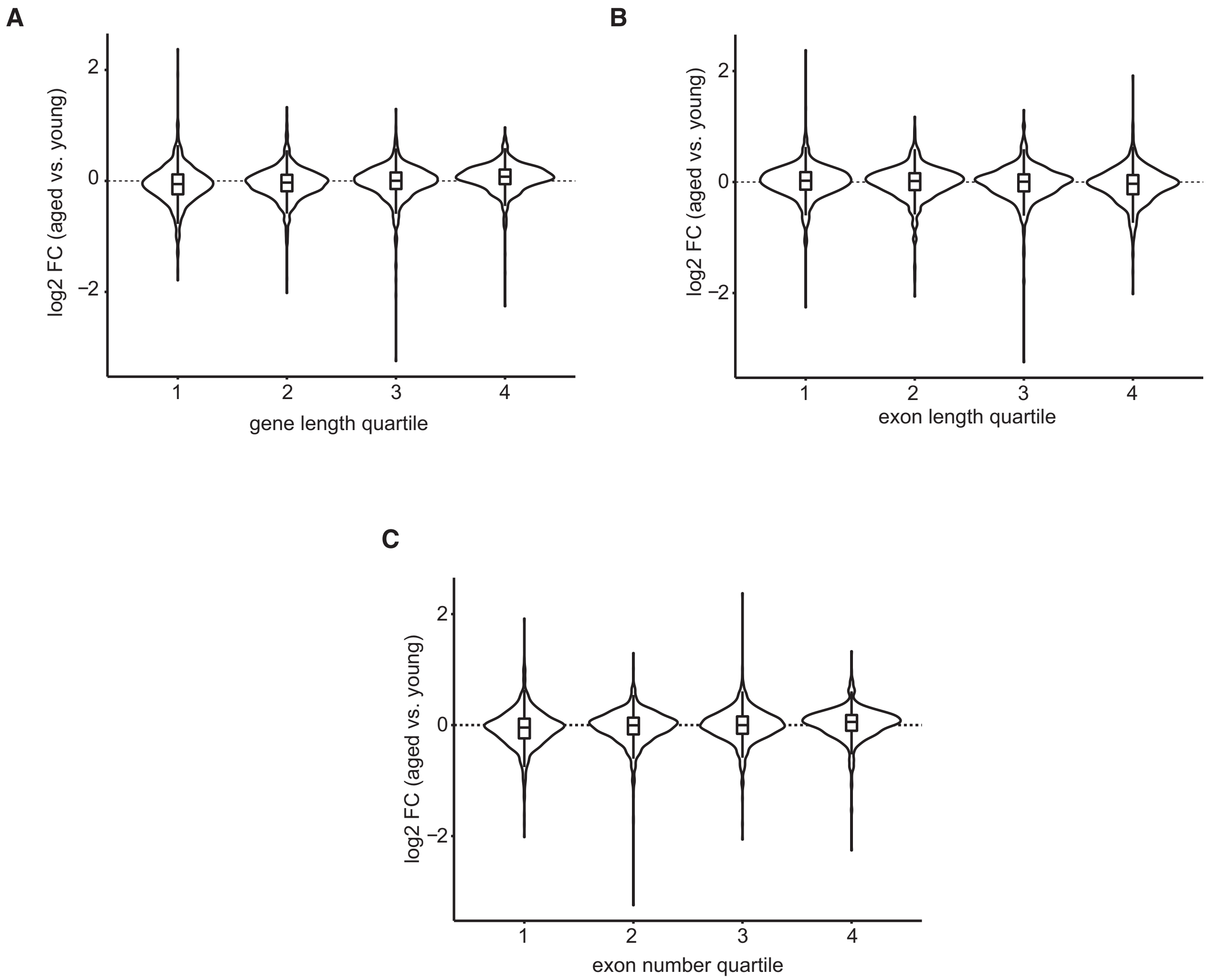

Figure EV3. Potential transcript features and impact on nascent transcription upon aging.

A–C Changes in nascent transcription (gene-body Pol II density, tNET-seq) in aged versus young animals in relationship to (A) gene length, (B) median exon length or (C) exon number per gene. The gene length was calculated as the total exonic length after reducing a gene's exons to a non-overlapping set. Number of genes per quartile: 819. Box plots consist of the median (central line), the 25th and 75th percentiles (box) and the highest/lowest value within 1.5 * interquartile range of the box (whiskers). n = 3 biological replicates per age group.

 © 2022 The Authors

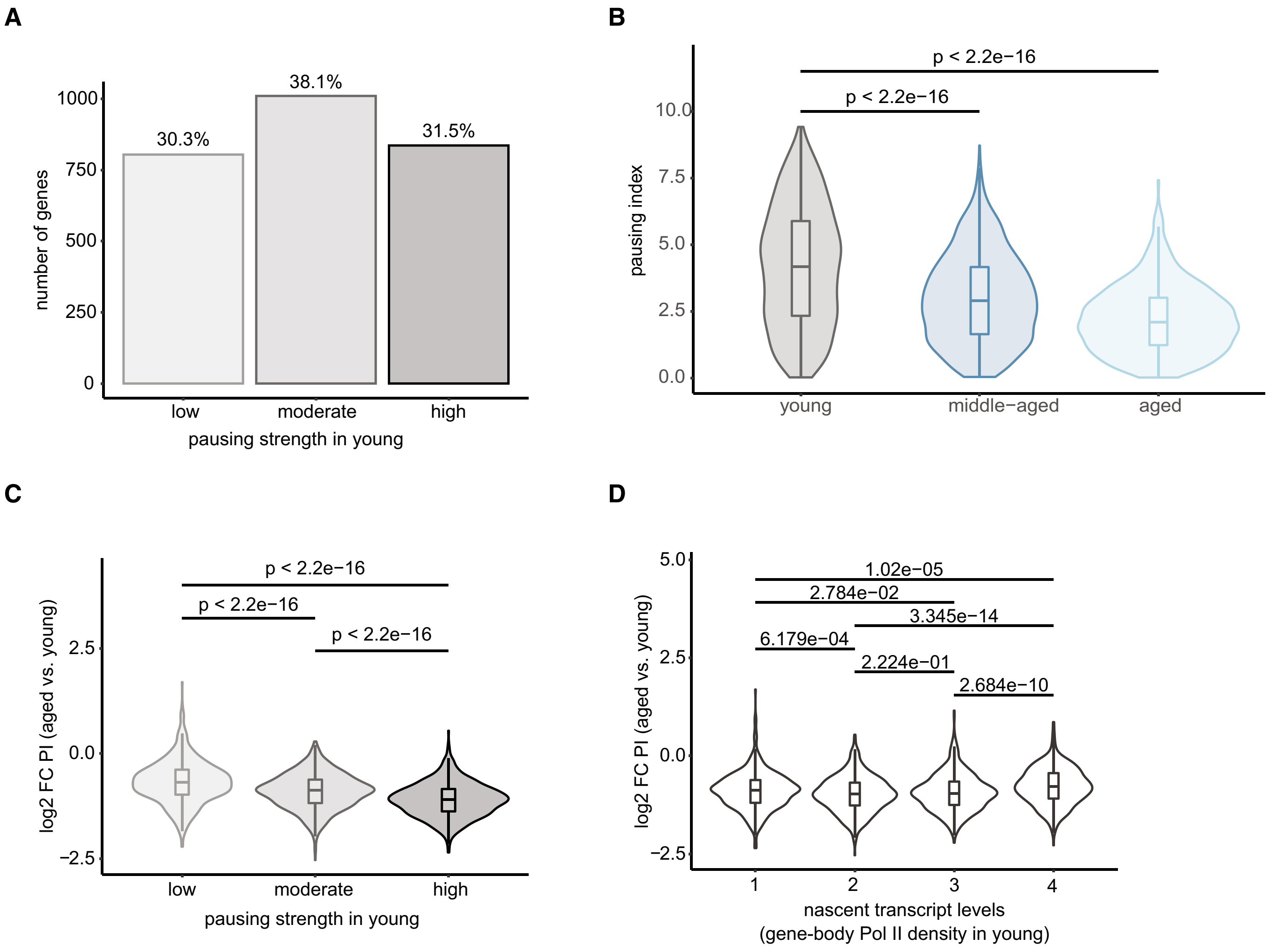

Figure EV4. RNA Pol II pausing characteristics.

A Bar plot of PIs quantifying the extent of promoter-proximal pausing in young mice. The 2,650 genes were divided into three groups based on the PI in young animals: highly paused (PI ≥ 3; *n* = 836), moderately paused (1.5 ≤ PI < 3; *n* = 1,010), and lowly paused (PI < 1.5; *n* = 804).

B Violin and box plots of PI values (based on TSS +20 to TSS +100 bp) in young, middle-aged and aged animals. *P*-values were calculated using a two-sided Wilcoxon rank-sum test (*n* = 1.289 genes). Box plots consist of the median (central line), the 25th and 75th percentiles (box) and the highest/lowest value within 1.5 * interquartile range of the box (whiskers).

C Violin and box plot of change in PI of aged versus young liver. Genes were grouped by PI as in (A).

D Violin and box plots of PI change in aged versus young animals. Genes were divided into four equal-sized groups based on their level of nascent transcription (gene-body Pol II density) in the liver of young mice (*n* = 2,650 genes). Box plots consist of the median (central line), the 25th and 75th percentiles (box) and the highest/lowest value within 1.5 * interquartile range of the box (whiskers). *P*-values were calculated using a two-sided Wilcoxon rank-sum test. Data information: *n* = 3 biological replicates per age group.

Figure EV5. Enhancers and TF abundance.

A Genomic distribution of identified active enhancers.

B Violin and boxplots of log2-fold changes in Pol II density at enhancer regions of middle-aged and aged animals relative to young ones (*n* = 8,144). Box plots consist of the median (central line), the 25th and 75th percentiles (box) and the highest/lowest value within 1.5 * interquartile range of the box (whiskers).

C Heatmap of steady-state mRNA levels (RNA-seq) of relevant transcription regulators in young, middle-aged and aged mice. Normalized read counts (rlog transformation, DESeq2) are reported.

D Heatmap of protein abundance of relevant transcription regulators in young and aged mice assessed by mass spectrometry. Normalized TMT reporter intensities (vsn-normalization, limma) are reported.

Data information: *n* = 3–4 biological replicates per age group for RNA-seq and tNET-seq; *n* = 4–5 for proteomics as indicated in the heatmaps.

© 2022 The Authors

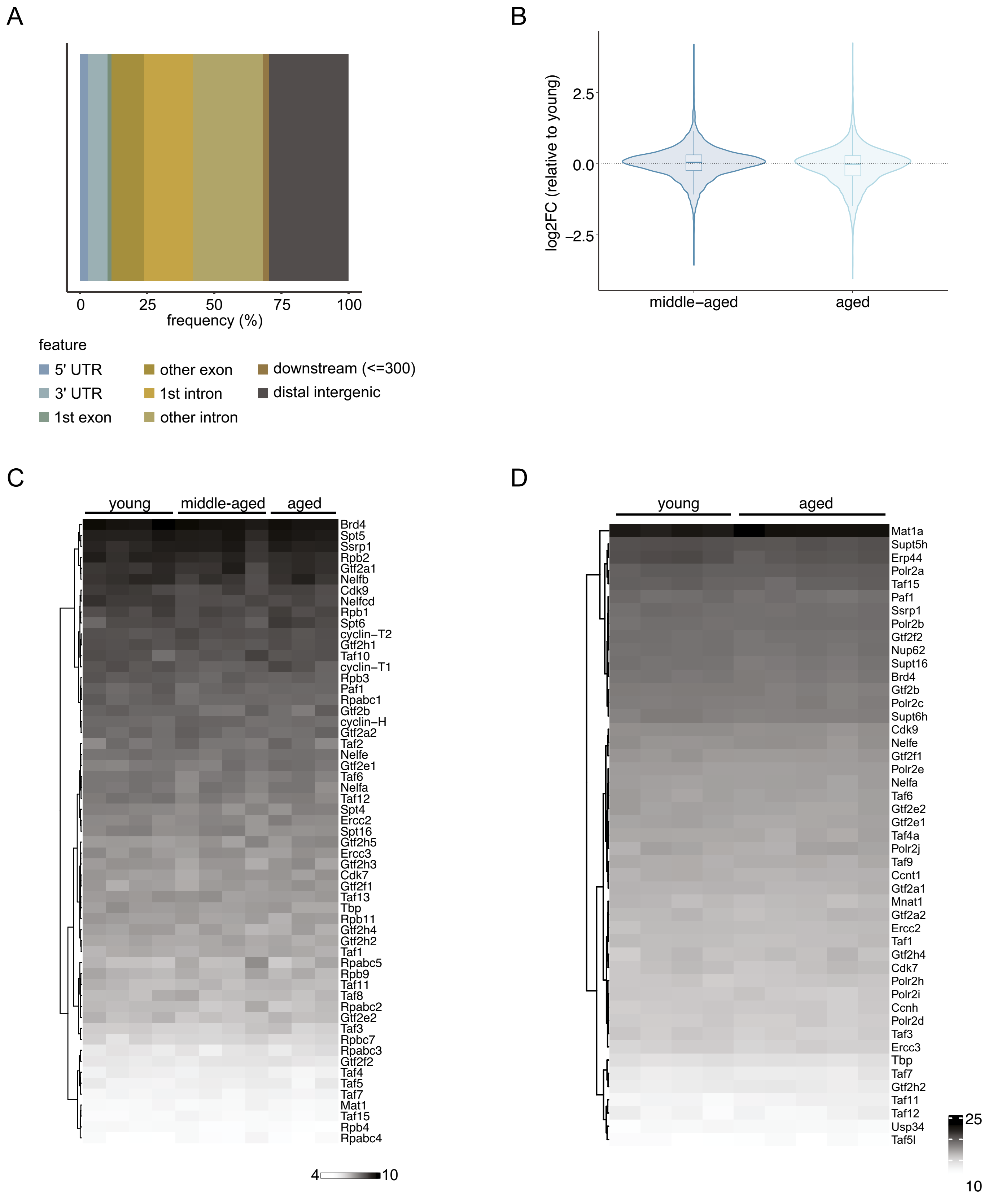

Figure EV5.

 © 2022 The Authors
