# Supplementary material for: Aging is associated with increased chromatin accessibility and reduced polymerase pausing in liver
Source: Mol Syst Biol. 2022 Sep 9;18(9):e11002. doi: 10.15252/msb.202211002 (PMC9459415; doi:10.15252/msb.202211002)
Supplement: Supplementary file 9 [file MSB-18-e11002-s007.pdf]

molecular systems biology

Aging is associated with increased chromatin accessibility and reduced polymerase pausing in liver

Mihaela Bozukova, Chrysa Nikopoulou, Niklas Kleinenkuhnen, Dora Grbavac, Katrin Goetsch, and Peter Tessarz
DOI: 10.15252/msb.202211002

Corresponding author(s): Peter Tessarz (ptessarz@age.mpg.de)

Review Timeline:

Submission Date:	25th Feb 22
Editorial Decision:	1st Apr 22
Revision Received:	19th Jul 22
Editorial Decision:	3rd Aug 22
Revision Received:	9th Aug 22
Accepted:	15th Aug 22

Editor: Maria Polychronidou

Transaction Report:

(Note: With the exception of the correction of typographical or spelling errors that could be a source of ambiguity, letters and reports are not edited. Depending on transfer agreements, referee reports obtained elsewhere may or may not be included in this compilation. Referee reports are anonymous unless the Referee chooses to sign their reports.)

1st Editorial Decision 1st Apr 2022

RE: MSB-2022-11002, Reduced polymerase pausing compensates for increased chromatin accessibility in the aging liver

Thank you again for submitting your work to Molecular Systems Biology. Unfortunately, despite several reminders, we have not received a report from reviewer #3. In the interest of time, and given that the recommendations of reviewers #1 and #2 are similar, I have now decided to proceed with making a decision based on the two available reports. If reviewer #3 sends us their report within the next few days, I will forward it to you separately so that you can also address their points. As you will see below, the two reviewers think that the study seems interesting. However, they raise a series of concerns, which we would ask you to address in a revision.

The comments of the referees are rather clear and seem straightforward to address so I think there is no need to repeat any of them here. All issues raised by the referees would need to be satisfactorily addressed. Please let me know in case you would like to discuss in further detail any of the issues raised, I would be happy to schedule a call.

On a more editorial level, we would ask you to address the following points:

Reviewer #1:

Here, Bozukova et al., apply and integrate several omic techniques to characterize age-related changes in chromatin organization and nascent transcription, using tissues from young, middle-aged and aged mice. This builds on other recent studies from this group on aging vs epigenetic changes. In addition a modified NET-seq protocol for tissues is described which may be highly useful to other researchers in the future. They detect a new and interesting compensatory mechanism to maintain transcriptional fidelity, and begin its investigation.
Overall, the study is well designed and executed, and provides a timely addition to the rapidly emerging field of transcriptional dysfunction with aging. I thank the Authors for an interesting manuscript that was easy to read.
Below, I make some major and minor comments. The Major comments refer to the interpretation of the data, and may be easily addressed.

Major Comments
1. Interpretation of NET-seq and pausing. The Authors refer to NET-seq throughout the manuscript as a proxy of nascent transcription. However, there is a crucial case in the vicinity of the promoter/TSS which should be clarified. NET-seq reflects nascent transcription at single bp resolution. In this way, nascent transcription and Pol II abundance are considered equivalent in the gene body and TES (Transcription End Site). However, at the promoter (and here the Authors use a window of the TSS+/-200bp (Transcription start site)), it could be argued that NET-seq more specifically represents RNA Pol II abundance, since the Pol II may be static at the +1 site, or paused at ~ +30-60bp. So a window of TSS+/-200bp is too large to distinguish between Pol II assembly, initiation, pausing, and elongation. While the current raw data is not in doubt to me, the interpretation of the following 2 concepts in the manuscript needs revision:
-NET-seq signal in the promoter region (as defined by the Authors: TSS+/-200bp) is not safe to assume as equivalent to paused Pol II. While the Authors can speculate that paused Pol II becomes altered by aging, an alternative could be that recruitment of Pol II is reduced around the +1 site. Similarly, while the Authors convincingly show less Spt4 abundance in the TSS +/-2000bp, this could be because there is less Pol II complex present. Is Spt4 selectively reduced compared to Pol II itself, or other components of the complex such as the Ptefb complex? The Authors could address this with further experiments to precisely assess Pol II assembly, initiation, pausing, and elongation, or clearly explain this limitation and tone down the interpretation on mechanism.
-Pol II pausing is interpreted in the manuscript as NET-seq signal (Pol II abundance) at the TSS+/-200bp in a ratio with the signal in the gene body > +200bp. Since the Authors use NET-seq which has single bp resolution, in order to selectively analyse Pol II pausing at +20 to +60bp, perhaps the Authors could restrict their analysis to the +20 to +100bp region of the genes, in order to distinguish it from Pol II which is static and assembling at +1 or elongating at > +100bp. Do the same relationships emerge for PI (Pausing Index) when paused Pol II abundance is considered this way?

2. Interpretation of the mechanism. The Authors observe increased chromatin accessibility at the promoter coincides with reduced Pol II paused complex (or Pol II abundance, as discussed above). The Authors interpret the observations as a compensatory mechanism to maintain transcription fidelity during aging (Lines 28-29 of Abstract; also in Discussion, 484-490). This appears to imply that evolution has selected for a compensation process against aging, which would be unusual in a post-reproductive old individual. Is it necessary to associate this with old age? An alternative explanation could be a fidelity mechanism to promote survival in youth, for example after disruption by physical or transient damage signals, which could become mal-adaptive in old cells. A case of antagonistic pleiotropy. In old cells, persistent aberrant input signals could persistently trigger the same mechanism, leading to dysfunction. In my opinion, the Authors imply natural selection to compensate against aging, and this is difficult to reconcile, and perhaps not required. Such a compensatory mechanism to maintain transcription fidelity could evolve to operate routinely and transiently in young cells and tissues. This would still be an important mechanism for the field to further investigate.
3. A central point in the manuscript is that chromatin accessibility is increased in the promoter region of old cells. Since it is a central point, could the Authors validate this by another technique, for example, at 3-4 genes.

Minor Comments
1. Could the Authors please provide their definition of the TES site used?
2. ATAC-seq changes occur at 8% of sites. The Authors note that more sites change in ATC-seq than in the RNA-seq - to highlight this, could the Authors indicate, in the relevant Results text, the % of total genes that are differentially-expressed with a similar threshold in young vs old by RNAs-seq?
3. The data points are very small and/or the line colours are difficult to distinguish (Examples: Figures: 2C, 4A, S1E, S3A, S4C).
4. In Figure 3D the Authors note a cluster of genes that shows an average change in nascent transcription that appears decreased in middle-aged and old-aged mice (relative to young). However, the associated text describes that the cluster contains inflammatory genes, which might be expected to increase (Lines: 238-241) -could the Authors please clarify this part?
5. Line 282-287: The Authors suggest that the Pausing Index (PI) is >1.5x in 69.7% of genes, and that similar levels of PI have been reported -can the Authors provide a reference for this? Several studies report a PI of >2x in ~90% of genes. If there is a difference in their study, can the Authors comment on that in the text?
6. Could the Author confirm the p-Values in some Figures. The value of 2,2 x 10-16 appears 6 times -is this correct? (Figures: 1D, 4B(twice), S2A, S7B(twice)

Reviewer #2:

Reduced polymerase pausing compensates for increased chromatin accessibility in the ageing liver, Bozukova et al., Tessarz lab.
How ageing affects tissue and cellular processes at the molecular level is an important question.
In this study, the author show that ageing in mouse liver is characterized by an increase in chromatin accessibility at promoter regions. This is not accompanied by an increase of transcriptional output but by a decrease of promoter-proximal pausing of RNA polymerase II (Pol II). This decrease is linked to an decreased binding of factor SPT4, which promotes PolII pausing as a component of the pause-inducing factor DSIF. The authors then propose that these changes in transcriptional regulation are due to a reduced stability of the pausing complex and may represent a mechanism to compensate for the age-related increase in chromatin accessibility in order to prevent aberrant transcription, mitigating the effects of the overall increase in promoter accessibility to maintain a relatively young-like transcription state in the ageing liver.
The authors also conclude, based on the comparison of changes in nascent transcription and steady-state transcriptom, that many processes, such as mRNA processing and degradation shape the coordinated gene expression signature detected in liver.

The study is well done and the data are of high quality and reveal a new aspect of regulation that can be linked to ageing. The tNET-seq methodology will be valuable for many researchers in the fields of gene and genome regulation. There are some limitations of the study, some simply reflect the problems linked to study ageing in the mouse, where mechanistic/ intervention studies will take many years. However, the authors can improve the study considering following points:

1.) While the authors observed a decreased occupancy of pausing regulator SPT4 at TSSs of tNET genes in aged animals, without more mechanistic studies, the link between changes in chromatin accessibility of promoters on ageing and changes in RNA polymerase pausing is somewhat weak. The authors do not provide evidence for the negative feedback loop they propose, (their model that "increased chromatin accessibility on ageing, a transient increase in transcription leading to a change in "stability of the pausing complex to prevent further aberrant transcription and maintain transcription fidelity"). Therefore, this speculation should be weakened or taken out (e.g., from abstract) without further data and alternative explanations should be considered. Changes in accessibility may be linked to function of (e.g. chromatin remodelling) factors that, in turn, also regulate SPT4 binding and RNA polymerase pausing (see for example: DOI: 10.1186/s13059-021-02500-1). So, there is a 'chicken or egg' problem.

2.) The critical question is: what is changed on the Polymerase II pausing regulating machinery on ageing and why? As a minimum, the authors should evaluate cellular levels of SPT4 protein in young versus aged (and other DSIF components). Drop in (active) SPT4 protein levels may explain loss of its promoter occupancy.

3.) There is no description of how ageing affects the mice and liver function in the aged mouse colony. Are the old mice obese? Do the aged mice exhibit liver steatosis? What are the changes in cellular composition of the livers of the aged versus young mice. This is important to show for their aged mouse colony, as every aged mouse colony is different. The authors show that the aged livers exhibit changes in gtranscription linked to metabolism and there is transcriptional evidence for inflammaging. It would be good to put this study into a stronger biological context.

Minor comments:
While the number of animals used for some analysis (e.g., ATAC-seq) is stated in the methods, this seems not be clear for all. For transparency, this should be stated in each figure.

The writing needs to be more precise and explicit, avoiding shorthands that might have been useful in the lab, e.g., what are "tNET promoters or enhancers" (page 15, line 385) tNET genes etc etc. This gets sometimes really confusing, e.g. (line 252): "We found 34 % of these aging genes (396 out of 1,158) to be differentially transcribed with age, which is in the same order of magnitude as the overall differentially transcribed genes 259 (29 %, 953 out of 3,278)" What is what here?

As the NET-seq method is central to this study, the authors should outline the principle of the method in the result section.

The authors used a published data set for steady-state transcriptom and compare this with their nascent gene expression analysis. This leaves me somewhat uncomfortable, as gene expression may differ from one aged mouse colony to another. The analysis would be more convincing if they had performed RNA-seq and tNET-seq in parallel.

We would like to thank the reviewers for the positive statements and the constructive criticism that certainly helped to improve our manuscript. We have edited the text and added new data following your suggestions. Please find our point-by-point response below. To facilitate re-review, we have highlighted the changes in the revised manuscript text in yellow. We hope that – like us - you'll find the revised version of the manuscript improved and support publication.

Reviewer #1:

Here, Bozukova et al., apply and integrate several omic techniques to characterize age-related changes in chromatin organization and nascent transcription, using tissues from young, middle-aged and aged mice. This builds on other recent studies from this group on aging vs epigenetic changes. In addition a modified NET-seq protocol for tissues is described which may be highly useful to other researchers in the future. They detect a new and interesting compensatory mechanism to maintain transcriptional fidelity, and begin its investigation.
Overall, the study is well designed and executed, and provides a timely addition to the rapidly emerging field of transcriptional dysfunction with aging. I thank the Authors for an interesting manuscript that was easy to read.

We thank the reviewer for the encouraging words on our manuscript!

Below, I make some major and minor comments. The Major comments refer to the interpretation of the data, and may be easily addressed.

Major Comments
1. Interpretation of NET-seq and pausing. The Authors refer to NET-seq throughout the manuscript as a proxy of nascent transcription. However, there is a crucial case in the vicinity of the promoter/TSS which should be clarified. NET-seq reflects nascent transcription at single bp resolution. In this way, nascent transcription and Pol II abundance are considered equivalent in the gene body and TES (Transcription End Site). However, at the promoter (and here the Authors use a window of the TSS+/-200bp (Transcription start site)), it could be argued that NET-seq more specifically represents RNA Pol II abundance, since the Pol II may be static at the +1 site, or paused at ~ +30-60bp. So a window of TSS+/-200bp is too large to distinguish between Pol II assembly, initiation, pausing, and elongation. While the current raw data is not in doubt to me, the interpretation of the following 2 concepts in the manuscript needs revision:

-NET-seq signal in the promoter region (as defined by the Authors: TSS+/-200bp) is not safe to assume as equivalent to paused Pol II. While the Authors can speculate that paused Pol II becomes altered by aging, an alternative could be that recruitment of Pol II is reduced around the +1 site. Similarly, while the Authors convincingly show less Spt4 abundance in the TSS +/-2000bp, this could be because there is less Pol II complex present. Is Spt4 selectively reduced compared to Pol II itself, or other components of the complex such as the Ptefb complex? The Authors could address this with further experiments to precisely assess Pol II assembly, initiation, pausing, and elongation, or clearly explain this limitation and tone down the interpretation on mechanism.

We agree with the reviewer that more directly addressing Pol II initiation and assembly would strengthen the conclusions of the manuscript. Thus, we performed CUT&RUN for RNA Pol II as well as RNA Pol II S2 and S5 phospho to assess RNA Pol II loading, initiation and elongation. These experiments confirmed our previous observations that RNA Pol II loading and initiation are not affected by ageing – if at all, we observe a tendency for an increase at higher age, which

would be in line with an increase in chromatin accessibility. RNA Pol II S2 phospho levels do not change between young and aged liver, supporting the nascent RNA data showing no difference in elongation. These data are included as new Figures 5E and the corresponding text can be found in lines 341-345 and 445-446.

-Pol II pausing is interpreted in the manuscript as NET-seq signal (Pol II abundance) at the TSS+/-200bp in a ratio with the signal in the gene body > +200bp. Since the Authors use NET-seq which has single bp resolution, in order to selectively analyse Pol II pausing at +20 to +60bp, perhaps the Authors could restrict their analysis to the +20 to +100bp region of the genes, in order to distinguish it from Pol II which is static and assembling at +1 or elongating at > +100bp. Do the same relationships emerge for PI (Pausing Index) when paused Pol II abundance is considered this way?

We understand this concern. Usually, the window for calculating the pausing index is taken more narrowly. We initially decided to take a wider window in order to be consistent with the one chosen for ATAC-seq. To make our data more comparable to published reports on promoter proximal pausing, we have repeated our analysis for the interval [TSS + 20 bp; TSS + 100 bp]. The results in terms of Pol II density and pausing index are in line with the wider TSS window and confirm the age-related decrease in promoter-proximal pausing. The violin-boxplot of the narrow window that matches the wider one we included in the first version of the manuscript has been included as new Figure EV4B.

2. Interpretation of the mechanism. The Authors observe increased chromatin accessibility at the promoter coincides with reduced Pol II paused complex (or Pol II abundance, as discussed above). The Authors interpret the observations as a compensatory mechanism to maintain transcription fidelity during aging (Lines 28-29 of Abstract; also in Discussion, 484-490). This appears to imply that evolution has selected for a compensation process against aging, which would be unusual in a post-reproductive old individual. Is it necessary to associate this with old age? An alternative explanation could be a fidelity mechanism to promote survival in youth, for example after disruption by physical or transient damage signals, which could become mal-adaptive in old cells. A case of antagonistic pleiotropy. In old cells, persistent aberrant input signals could persistently trigger the same mechanism, leading to dysfunction. In my opinion, the Authors imply natural selection to compensate against aging, and this is difficult to reconcile, and perhaps not required. Such a compensatory mechanism to maintain transcription fidelity could evolve to operate routinely and transiently in young cells and tissues. This would still be an important mechanism for the field to further investigate.

This is an interesting point. By no means, we wanted to start a discussion on an evolutionary aspect of ageing as we do not think our data is broad enough for such a claim. The reason we believe it is a compensatory mechanism is the fact that in ageing liver few genes are differentially expressed, particularly given the large changes on the level of chromatin. However, we agree that this is – at the moment – pure speculation and other reasons might be responsible for the effect we observe. As also reviewer 2 commented on this issue, we have weakened our claim by removing the idea of compensation from the title and abstract, and clearly point out in the results (line 362) and discussion that this is speculation at the moment and added potentially other reasons for our observations (lines 448-452): *“Future work will have to address the upstream events that lead to these age-related changes, whether they represent a compensation for the increase in promoter accessibility in order to maintain a balanced transcriptome, altered signaling events or changes in the efficiency of chromatin remodelling.”*

3. A central point in the manuscript is that chromatin accessibility is increased in the promoter region of old cells. Since it is a central point, could the Authors validate this by another technique, for example, at 3-4 genes.

We agree with the reviewer that it would be good to test the observed increase in accessibility using an alternative approach. We used a previously published dataset that mapped nucleosome positioning by MNase-seq in the aging liver as an alternative dataset (PMID: 25437555). Similar to our ATAC-seq, we observed a global loss of nucleosomes (increase in accessibility) at promoter regions with age, which can also be monitored on a gene-by-gene case. This new analysis is available as Appendix Figure S3 in the revised version of the manuscript.

Minor Comments

1. Could the Authors please provide their definition of the TES site used?

Clarified in Materials and Methods, section “Gene selection” (lines 717-726).

2. ATAC-seq changes occur at 8% of sites. The Authors note that more sites change in ATC-seq than in the RNA-seq - to highlight this, could the Authors indicate, in the relevant Results text, the % of total genes that are differentially-expressed with a similar threshold in young vs old by RNAs-seq?

Added in results, section “Aging has a modest effect on the transcriptional output” (lines 155-157).

3. The data points are very small and/or the line colours are difficult to distinguish (Examples: Figures: 2C, 4A, S1E, S3A, S4C).

We have now made the lines thicker and hope that they are now more distinguishable.

4. In Figure 3D the Authors note a cluster of genes that shows an average change in nascent transcription that appears decreased in middle-aged and old-aged mice (relative to young). However, the associated text describes that the cluster contains inflammatory genes, which might be expected to increase (Lines: 238-241) -could the Authors please clarify this part?

This is such a small cluster that made it impossible to perform GO enrichment (clarified this point now in the revised manuscript, lines 239-240). The few genes involved in interferon response also do not have a clear-cut phenotypes and there are reports in the literature, e.g. Nlrc5 has been reported to have anti-fibrogenic functions (PMID: 26806094). If the reviewer thinks that this part is too confusing, we would be happy to take out the reference to inflammation.

5. Line 282-287: The Authors suggest that the Pausing Index (PI) is >1.5x in 69.7% of genes, and that similar levels of PI have been reported -can the Authors provide a reference for this? Several studies report a PI of >2x in ~90% of genes. If there is a difference in their study, can the Authors comment on that in the text?

We have added a reference in the results section “Promoter-proximal Pol II pausing decreased with age” (line 276). We have also re-analyzed using PI >2 as cut-off. In this case, 54.8 % of genes (1,453 out of 2,650) included in our analysis exhibit a PI > 2. Due to the broad range of pausing indices among genes and the difficulty of applying discrete cut-offs to such a continuum,

studies have reported estimates ranging from 30 to 90% of paused genes for multiple human and mouse cell types PMID: 22986266). This broad range is most likely a consequence of different experimental methods (ChIP-seq vs. nascent methods such as PRO- and NET-seq) and statistical criteria used to define Pol II pausing. Within this broad range, previous studies in HepG2 cells and mouse liver tissue found ~35% and ~50% of genes to exhibit a PI > 2 (PMID: 27259512), in line with our results. We chose a more fine-grained pausing classification, distinguishing between lowly, moderately and highly paused genes to investigate how these different pausing classes are affected by age. A study in bone marrow-derived macrophages using the same pausing classification found 76% (ChIP-seq) or 83% (PRO-seq) of genes to be highly or moderately paused, which is in a similar range as our results.

6. Could the Author confirm the p-Values in some Figures. The value of 2,2 x 10-16 appears 6 times -is this correct? (Figures: 1D, 4B(twice), S2A, S7B(twice)

The programing language R uses this notation automatically for reporting very small numbers, as 2.2e-16 is the smallest number larger than 0 that can be stored by the floating system due to computer precision limits.

Reviewer #2:

Reduced polymerase pausing compensates for increased chromatin accessibility in the ageing liver, Bozukova et al., Tessarz lab.
How ageing affects tissue and cellular processes at the molecular level is an important question. In this study, the author show that ageing in mouse liver is characterized by an increase in chromatin accessibility at promoter regions. This is not accompanied by an increase of transcriptional output but by a decrease of promoter-proximal pausing of RNA polymerase II (Pol II). This decrease is linked to an decreased binding of factor SPT4, which promotes PolII pausing as a component of the pause-inducing factor DSIF. The authors then propose that these changes in transcriptional regulation are due to a reduced stability of the pausing complex and may represent a mechanism to compensate for the age-related increase in chromatin accessibility in order to prevent aberrant transcription, mitigating the effects of the overall increase in promoter accessibility to maintain a relatively young-like transcription state in the ageing liver.
The authors also conclude, based on the comparison of changes in nascent transcription and steady-state transcriptom, that many processes, such as mRNA processing and degradation shape the coordinated gene expression signature detected in liver.

The study is well done and the data are of high quality and reveal a new aspect of regulation that can be linked to ageing. The tNET-seq methodology will be valuable for many researchers in the fields of gene and genome regulation.

We thank the reviewer for the positive comment on our manuscript.

There are some limitations of the study, some simply reflect the problems linked to study ageing in the mouse, where mechanistic/ intervention studies will take many years. However, the authors can improve the study considering following points:

1.) While the authors observed a decreased occupancy of pausing regulator SPT4 at TSSs of tNET genes in aged animals, without more mechanistic studies, the link between changes in chromatin accessibility of promoters on ageing and changes in RNA polymerase pausing is somewhat weak. The authors do not provide evidence for the negative feedback loop they

propose, (their model that "increased chromatin accessibility on ageing, a transient increase in transcription leading to a change in "stability of the pausing complex to prevent further aberrant transcription and maintain transcription fidelity"). Therefore, this speculation should be weakened or taken out (e.g., from abstract) without further data and alternative explanations should be considered. Changes in accessibility may be linked to function of (e.g. chromatin remodelling) factors that, in turn, also regulate SPT4 binding and RNA polymerase pausing (see for example: DOI: 10.1186/s13059-021-02500-1). So, there is a 'chicken or egg' problem.

We agree with this reviewer (and reviewer 1) that the model is speculative at the moment. We have tried and make this clearer in the revised version of the manuscript, e.g. we have taken out the idea of compensation from the title and abstract and have labelled it as speculation in the results (line 362) and discussion (lines 448-452). We have also added a section in the discussion, in which we have included other potential explanations for our observation (lines 448-452).

2.) The critical question is: what is changed on the Polymerase II pausing regulating machinery on ageing and why? As a minimum, the authors should evaluate cellular levels of SPT4 protein in young versus aged (and other DSIF components). Drop in (active) SPT4 protein levels may explain loss of its promoter occupancy.

We agree with the reviewer that this is an important question, which we would really have liked to address. However, as noted by the reviewer, this is difficult to achieve in an aging model. To start addressing this point, we followed the suggestion of the reviewer and have now performed whole tissue proteomics to analyze the levels of transcription initiation/elongation factors. Similar to the RNA-seq data, we do not observe any differences in the level of these factors. We have included this dataset as new Figure EV5D and Table EV6.

3.) There is no description of how ageing affects the mice and liver function in the aged mouse colony. Are the old mice obese? Do the aged mice exhibit liver steatosis? What are the changes in cellular composition of the livers of the aged versus young mice. This is important to show for their aged mouse colony, as every aged mouse colony is different. The authors show that the aged livers exhibit changes in gtranscription linked to metabolism and there is transcriptional evidence for inflammaging. It would be good to put this study into a stronger biological context.

We use physiologically healthy individuals that show no signs of fibrosis, inflammation and/or pathological steatosis. We have added this information also in the Materials and Methods section (line 461-464). To illustrate this point, we have now added a representative H&E stain of young and old liver section (new Appendix Figure S1). With respect to the changes in cellular composition, we would refer the reviewer to a recent manuscript from our laboratory, in which we performed single-cell analysis of aging liver (https://www.biorxiv.org/content/10.1101/2021.12.14.472593v1), in which we do not see major remodelling of liver tissue with respect to cell composition. We have now added this information to the discussion (lines 391-392).

Minor comments:

While the number of animals used for some analysis (e.g., ATAC-seq) is stated in the methods, this seems not be clear for all. For transparency, this should be stated in each figure.

We have added this information to all figures.

The writing needs to be more precise and explicit, avoiding shorthands that might have been useful in the lab, e.g., what are "tNET promoters or enhancers" (page 15, line 385) tNET genes etc etc. This gets sometimes really confusing, e.g. (line 252): "We found 34 % of these aging genes (396 out of 1,158) to be differentially transcribed with age, which is in the same order of magnitude as the overall differentially transcribed genes 259 (29 %, 953 out of 3,278)" What is what here?

We have now better defined tNET genes (definition, line 192). We would like to stick with this term as it clearly defines a subset of genes used in the analysis. We think that avoiding the name “tNET genes” altogether would be more confusing. We also refined the legend of Fig. 5 to make this more understandable and rephrased the paragraph regarding aging genes (lines 245-249). We hope these changes make it clearer now.

As the NET-seq method is central to this study, the authors should outline the principle of the method in the result section.

Added in results, section “Aging has a modest effect on the transcriptional output” (lines 170-179).

The authors used a published data set for steady-state transcriptom and compare this with their nascent gene expression analysis. This leaves me somewhat uncomfortable, as gene expression may differ from one aged mouse colony to another. The analysis would be more convincing if they had performed RNA-seq and tNET-seq in parallel.

We agree with the reviewer. To address this point in the scope of a revision, we used an RNA-seq we generated from our standing mouse aging colony (but that only consisted of young vs. old and did not have the middle-aged time point). The results support the notion that the overlap between nascent and steady-state transcription is minimal (new Figure EV2G-I).

RE: MSB-2022-11002R, Aging is associated with increased chromatin accessibility and reduced polymerase pausing in liver

Thank you for sending us your revised manuscript. We have now heard back from the two reviewers who were asked to evaluate your revised study. As you will see below, both reviewers are satisfied with the modifications made and are supportive of publication. Reviewer #1 only lists some minor issues, which we would ask you to fix in a final round of revision. We would also ask you to address a few minor editorial issues listed below.

Reviewer #1:

Reviewer 1: response to Author_Rebuttal_1
Reviewer 1: I thank the Authors for considering my comments carefully. Below, I reproduce the point-by-point rebuttal from the Authors, and add my new comments.

Reviewer 1: Major Comments

Reviewer 1: 1. Interpretation of NET-seq and pausing. The Authors refer to NET-seq throughout the manuscript as a proxy of nascent transcription. However, there is a crucial case in the vicinity of the promoter/TSS which should be clarified. NET-seq reflects nascent transcription at single bp resolution. In this way, nascent transcription and Pol II abundance are considered equivalent in the gene body and TES (Transcription End Site). However, at the promoter (and here the Authors use a window of the TSS+/-200bp (Transcription start site)), it could be argued that NET-seq more specifically represents RNA Pol II abundance, since the Pol II may be static at the +1 site, or paused at ~ +30-60bp. So a window of TSS+/-200bp is too large to distinguish between Pol II assembly, initiation, pausing, and elongation. While the current raw data is not in doubt to me, the interpretation of the following 2 concepts in the manuscript needs revision:
-NET-seq signal in the promoter region (as defined by the Authors: TSS+/-200bp) is not safe to assume as equivalent to paused Pol II. While the Authors can speculate that paused Pol II becomes altered by aging, an alternative could be that recruitment of Pol II is reduced around the +1 site. Similarly, while the Authors convincingly show less Spt4 abundance in the TSS +/- 2000bp, this could be because there is less Pol II complex present. Is Spt4 selectively reduced compared to Pol II itself, or other components of the complex such as the Ptefb complex? The Authors could address this with further experiments to precisely assess Pol II assembly, initiation, pausing, and elongation, or clearly explain this limitation and tone down the interpretation on mechanism.

Authors: We agree with the reviewer that more directly addressing Pol II initiation and assembly would strengthen the conclusions of the manuscript. Thus, we performed CUT&RUN for RNA Pol II as well as RNA Pol II S2 and S5 phospho to assess RNA Pol II loading, initiation and elongation. These experiments confirmed our previous observations that RNA Pol II loading and initiation are not affected by ageing - if at all, we observe a tendency for an increase at higher age, which would be in line with an increase in chromatin accessibility. RNA Pol II S2 phospho levels do not change between young and aged liver, supporting the nascent RNA data showing no difference in elongation. These data are included as new Figures 5E and the corresponding text can be found in lines 341-345 and 445-446
-Reviewer 1: Pol II pausing is interpreted in the manuscript as NET-seq signal (Pol II abundance) at the TSS+/- 200bp in a ratio with the signal in the gene body > +200bp. Since the Authors use NET-seq which has single bp resolution, in order to selectively analyse Pol II pausing at +20 to +60bp, perhaps the Authors could restrict their analysis to the +20 to +100bp region of the genes, in order to distinguish it from Pol II which is static and assembling at +1 or elongating at > +100bp. Do the same relationships emerge for PI (Pausing Index) when paused Pol II abundance is considered this way?

Authors: We understand this concern. Usually, the window for calculating the pausing index is taken more narrowly. We initially decided to take a wider window in order to be consistent with the one chosen for ATAC-seq. To make our data more comparable to published reports on promoter proximal pausing, we have repeated our analysis for the interval [TSS + 20 bp; TSS + 100 bp]. The results in terms of Pol II density and pausing index are in line with the wider TSS window and confirm the age-related decrease in promoter-proximal pausing. The violin-boxplot of the narrow window that matches the wider one we included in the first version of the manuscript has been included as new Figure EV4B.

Reviewer 1: The CUT+ RUN data with abundance of RNA Pol II Ser 5P and Ser2P is a good supporting evidence for the central claim regarding Pol II assembly, pausing and initiation. However it also requires the data that the Authors have now also produced with their tNETseq analysis at the TSS+20 to +100bp window. I think these additional data together are now quite convincing. I would only suggest that the Authors include this new analysis (TSS +20 to +100bp; tNETseq) in a main Figure (currently it is: new Fig EV4B), as it is very supportive of one of their major points.
Line 351(revised manuscript): Possible typo: "RNA loading" should be "RNA Pol II loading"?

Reviewer 1: 2. Interpretation of the mechanism. The Authors observe increased chromatin accessibility at the promoter coincides with reduced Pol II paused complex (or Pol II abundance, as discussed above). The Authors interpret the observations as a compensatory mechanism to maintain transcription fidelity during aging (Lines 28-29 of Abstract; also in Discussion, 484-490). This appears to imply that evolution has selected for a compensation process against aging, which would be unusual in a post-reproductive old individual. Is it necessary to associate this with old age? An alternative explanation could be a fidelity mechanism to promote survival in youth, for example after disruption by physical or transient damage signals, which could become mal- adaptive in old cells. A case of antagonistic pleiotropy. In old cells, persistent aberrant input signals could persistently trigger the same mechanism, leading to dysfunction. In my opinion, the Authors imply natural selection to compensate against aging, and this is difficult to reconcile, and perhaps not required. Such a compensatory mechanism to maintain transcription fidelity could evolve to operate routinely and transiently in young cells and tissues. This would still be an important mechanism for the field to further investigate.

Authors: This is an interesting point. By no means, we wanted to start a discussion on an evolutionary aspect of ageing as we do not think our data is broad enough for such a claim. The reason we believe it is a compensatory mechanism is the fact that in ageing liver few genes are differentially expressed, particularly given the large changes on the level of chromatin. However, we agree that this is - at the moment - pure speculation and other reasons might be responsible for the effect we observe. As also reviewer 2 commented on this issue, we have weakened our claim by removing the idea of compensation from the title and abstract, and clearly point out in the results (line 362) and discussion that this is speculation at the moment and added potentially other reasons for our observations (lines 448-452): "Future work will have to address the upstream events that lead to these age-related changes, whether they represent a compensation for the increase in promoter accessibility in order to maintain a balanced transcriptome, altered signaling events or changes in the efficiency of chromatin remodelling.

Reviewer 1: The Authors have addressed this concern very well and the new changes to the Text are clear.

Reviewer 1: 3. A central point in the manuscript is that chromatin accessibility is increased in the promoter region of old cells. Since it is a central point, could the Authors validate this by another technique, for example, at 3-4 genes.

Authors: We agree with the reviewer that it would be good to test the observed increase in accessibility using an alternative approach. We used a previously published dataset that mapped nucleosome positioning by MNase-seq in the aging liver as an alternative dataset (PMID: 25437555). Similar to our ATAC-seq, we observed a global loss of nucleosomes (increase in accessibility) at promoter regions with age, which can also be monitored on a gene-by-gene case. This new analysis is available as Appendix Figure S3 in the revised version of the manuscript.

Reviewer 1: Fully addressed. These new independent analyses provided by the Authors are excellent supporting evidence for the increase in promoter accessibility observed upon aging in the mouse liver.

Reviewer 1: Minor Comments

Reviewer 1: 1. Could the Authors please provide their definition of the TES site used?

Authors: Clarified in Materials and Methods, section "Gene selection" (lines 717-726).

Reviewer 1: Could the Authors clarify the TES site in their study, in the form of the size-window used (eg: Transcription End Site +/200bp). This specific information will greatly assist future readers in reproducing and interpreting the data.

Reviewer 1: 2. ATAC-seq changes occur at 8% of sites. The Authors note that more sites change in ATC-seq than in the RNA-seq - to highlight this, could the Authors indicate, in the relevant Results text, the % of total genes that are differentially-expressed with a similar threshold in young vs old by RNAseq?

Authors: Added in results, section "Aging has a modest effect on the transcriptional output" (lines 155-157).

Reviewer 1: The Authors have updated this very clearly.

Reviewer 1: 3. The data points are very small and/or the line colours are difficult to distinguish (Examples: Figures: 2C, 4A, S1E, S3A, S4C).

Authors: We have now made the lines thicker and hope that they are now more distinguishable.

Reviewer 1: The Authors have updated this very clearly.

Reviewer 1: 4. In Figure 3D the Authors note a cluster of genes that shows an average change in nascent transcription that appears decreased in middle-aged and old-aged mice (relative to young). However, the associated text describes that the cluster contains inflammatory genes, which might be expected to increase (Lines: 238-241) -could the Authors please clarify this part?

Authors: This is such a small cluster that made it impossible to perform GO enrichment (clarified this point now in the revised manuscript, lines 239-240). The few genes involved in interferon response also do not have a clear-cut phenotypes and there are reports in the literature, e.g. Nlrc5 has been reported to have anti-fibrogenic functions (PMID: 26806094). If the reviewer thinks that this part is too confusing, we would be happy to take out the reference to inflammation.

Reviewer 1: Cluster 4 is the smallest group in the analysis, reflecting the fewest genes, and therefore perhaps of the least importance. While this Cluster 4 contains some inflammatory genes, as the Authors explain, the GO term of inflammation is not enriched. So it is a minor point, and the Authors can choose to keep it in or remove the reference to it. I just want to advise that to me it seemed counter-intuitive that inflammatory genes would go down with aging, and it somewhat distracted from the main point of this panel.

Reviewer 1: 5. Line 282-287: The Authors suggest that the Pausing Index (PI) is >1.5x in 69.7% of genes, and that similar levels of PI have been reported -can the
Authors provide a reference for this? Several studies report a PI of >2x in ~90% of genes. If there is a difference in their study, can the Authors comment on that in the text?

Authors: We have added a reference in the results section "Promoter-proximal Pol II pausing decreased with age" (line 276). We have also re-analyzed using PI >2 as cut-off. In this case, 54.8 % of genes (1,453 out of 2,650) included in our analysis exhibit a PI >2. Due to the broad range of pausing indices among genes and the difficulty of applying discrete cut-offs to such a continuum, studies have reported estimates ranging from 30 to 90% of paused genes for multiple human and mouse cell types PMID: 22986266). This broad range is most likely a consequence of different experimental methods (ChIP-seq vs. nascent methods such as PRO- and NET-seq) and statistical criteria used to define Pol II pausing. Within this broad range, previous studies in HepG2 cells and mouse liver tissue found ~35% and ~50% of genes to exhibit a PI > 2 (PMID: 27259512), in line with our results. We chose a more fine-grained pausing classification, distinguishing between lowly, moderately and highly paused genes to investigate how these different pausing classes are affected by age. A study in bone marrow-derived macrophages using the same pausing classification found 76% (ChIP-seq) or 83% (PRO-seq) of genes to be highly or moderately paused, which is in a similar range as our results

Reviewer 1: This is very well explained, thank-you.

Reviewer 1: 6. Could the Author confirm the p-Values in some Figures. The value of 2,2 x 10-16 appears 6 times -is this correct? (Figures: 1D, 4B(twice), S2A, S7B(twice)

Authors: The programing language R uses this notation automatically for reporting very small numbers, as 2.2e-16 is the smallest number larger than 0 that can be stored by the floating system due to

computer precision limits.

Reviewer 1: Thank-you, all is clear. I would only suggest to include this explanation somewhere in the Methods/Statistical section for the benefit of other readers.

Reviewer #2:

The authors did an outstanding job to address the reviewers' comments and improved the manuscript. I commend the authors for their careful and diligent work. I hope to see this published soon.

We thank the reviewers once again for their constructive criticism that improved this manuscript and appreciate their support for publication. We'll answer the remaining points below. The comments pertaining to revision round 1 by reviewer 1 are highlighted in italics.

Reviewer #1:

Reviewer 1: response to Author_Rebuttal_1
Reviewer 1: I thank the Authors for considering my comments carefully. Below, I reproduce the point-by-point rebuttal from the Authors, and add my new comments.

We thank the reviewer for appreciating our efforts in the revision process.

Reviewer 1: Major Comments

REVISION 1: Reviewer 1: 1. Interpretation of NET-seq and pausing. The Authors refer to NET-seq throughout the manuscript as a proxy of nascent transcription. However, there is a crucial case in the vicinity of the promoter/TSS which should be clarified. NET-seq reflects nascent transcription at single bp resolution. In this way, nascent transcription and Pol II abundance are considered equivalent in the gene body and TES (Transcription End Site). However, at the promoter (and here the Authors use a window of the TSS+/-200bp (Transcription start site)), it could be argued that NET-seq more specifically represents RNA Pol II abundance, since the Pol II may be static at the +1 site, or paused at ~ +30-60bp. So a window of TSS+/-200bp is too large to distinguish between Pol II assembly, initiation, pausing, and elongation. While the current raw data is not in doubt to me, the interpretation of the following 2 concepts in the manuscript needs revision:
-NET-seq signal in the promoter region (as defined by the Authors: TSS+/-200bp) is not safe to assume as equivalent to paused Pol II. While the Authors can speculate that paused Pol II becomes altered by aging, an alternative could be that recruitment of Pol II is reduced around the +1 site. Similarly, while the Authors convincingly show less Spt4 abundance in the TSS +/-2000bp, this could be because there is less Pol II complex present. Is Spt4 selectively reduced compared to Pol II itself, or other components of the complex such as the Ptefb complex? The Authors could address this with further experiments to precisely assess Pol II assembly, initiation, pausing, and elongation, or clearly explain this limitation and tone down the interpretation on mechanism.

Authors: We agree with the reviewer that more directly addressing Pol II initiation and assembly would strengthen the conclusions of the manuscript. Thus, we performed CUT&RUN for RNA Pol II as well as RNA Pol II S2 and S5 phospho to assess RNA Pol II loading, initiation and elongation. These experiments confirmed our previous observations that RNA Pol II loading and initiation are not affected by ageing - if at all, we observe a tendency for an increase at higher age, which would be in line with an increase in chromatin accessibility. RNA Pol II S2 phospho levels do not change between young and aged liver, supporting the nascent RNA data showing no difference in elongation. These data are included as new Figures 5E and the corresponding text can be found in lines 341-345 and 445-446
-Reviewer 1: Pol II pausing is interpreted in the manuscript as NET-seq signal (Pol II abundance) at the TSS+/- 200bp in a ratio with the signal in the gene body > +200bp. Since the Authors use NET-seq which has single bp resolution, in order to selectively analyse Pol II pausing at +20 to +60bp, perhaps the Authors could restrict their analysis to the +20 to +100bp region of the genes, in order to distinguish it from Pol II which is static and assembling at +1 or elongating at > +100bp. Do the same relationships emerge for PI (Pausing Index) when paused Pol II abundance is considered this way?

Authors: We understand this concern. Usually, the window for calculating the pausing index is taken more narrowly. We initially decided to take a wider window in order to be consistent with the one chosen for ATAC-seq. To make our data more comparable to published reports on promoter proximal pausing, we have repeated our analysis for the interval [TSS + 20 bp; TSS + 100 bp]. The results in terms of Pol II density and pausing index are in line with the wider TSS window and confirm the age-related decrease in promoter-proximal pausing. The violin-boxplot of the narrow window that matches the wider one we included in the first version of the manuscript has been included as new Figure EV4B.

Reviewer 1: The CUT+ RUN data with abundance of RNA Pol II Ser 5P and Ser2P is a good supporting evidence for the central claim regarding Pol II assembly, pausing and initiation. However it also requires the data that the Authors have now also produced with their tNETseq analysis at the TSS+20 to +100bp window. I think these additional data together are now quite convincing. I would only suggest that the Authors include this new analysis (TSS +20 to +100bp; tNETseq) in a main Figure (currently it is: new Fig EV4B), as it is very supportive of one of their major points.

Although we agree with the reviewer that this window is the standard in the field, we would like to keep it in the Extended View figure as we are afraid that having two very similar plots showing the same data confusing. The way it currently is structured highlights that the extended data is supporting evidence.

Line 351(revised manuscript): Possible typo: "RNA loading" should be "RNA Pol II loading"?

fixed

REVISION 1: Reviewer 1: 2. Interpretation of the mechanism. The Authors observe increased chromatin accessibility at the promoter coincides with reduced Pol II paused complex (or Pol II abundance, as discussed above). The Authors interpret the observations as a compensatory mechanism to maintain transcription fidelity during aging (Lines 28-29 of Abstract; also in Discussion, 484-490). This appears to imply that evolution has selected for a compensation process against aging, which would be unusual in a post-reproductive old individual. Is it necessary to associate this with old age? An alternative explanation could be a fidelity mechanism to promote survival in youth, for example after disruption by physical or transient damage signals, which could become mal- adaptive in old cells. A case of antagonistic pleiotropy. In old cells, persistent aberrant input signals could persistently trigger the same mechanism, leading to dysfunction. In my opinion, the Authors imply natural selection to compensate against aging, and this is difficult to reconcile, and perhaps not required. Such a compensatory mechanism to maintain transcription fidelity could evolve to operate routinely and transiently in young cells and tissues. This would still be an important mechanism for the field to further investigate.

Authors: This is an interesting point. By no means, we wanted to start a discussion on an evolutionary aspect of ageing as we do not think our data is broad enough for such a claim. The reason we believe it is a compensatory mechanism is the fact that in ageing liver few genes are differentially expressed, particularly given the large changes on the level of chromatin. However, we agree that this is - at the moment - pure speculation and other reasons might be responsible for the effect we observe. As also reviewer 2 commented on this issue, we have weakened our claim by removing the idea of compensation from the title and abstract, and clearly point out in the results (line 362) and discussion that this is speculation at the moment and added potentially

other reasons for our observations (lines 448-452): "Future work will have to address the upstream events that lead to these age-related changes, whether they represent a compensation for the increase in promoter accessibility in order to maintain a balanced transcriptome, altered signaling events or changes in the efficiency of chromatin remodelling.

Reviewer 1: The Authors have addressed this concern very well and the new changes to the Text are clear.

REVISION 1: Reviewer 1: 3. A central point in the manuscript is that chromatin accessibility is increased in the promoter region of old cells. Since it is a central point, could the Authors validate this by another technique, for example, at 3-4 genes.

Authors: We agree with the reviewer that it would be good to test the observed increase in accessibility using an alternative approach. We used a previously published dataset that mapped nucleosome positioning by MNase-seq in the aging liver as an alternative dataset (PMID: 25437555). Similar to our ATAC-seq, we observed a global loss of nucleosomes (increase in accessibility) at promoter regions with age, which can also be monitored on a gene-by-gene case. This new analysis is available as Appendix Figure S3 in the revised version of the manuscript.

Reviewer 1: Fully addressed. These new independent analyses provided by the Authors are excellent supporting evidence for the increase in promoter accessibility observed upon aging in the mouse liver.

Reviewer 1: Minor Comments

Reviewer 1: 1. Could the Authors please provide their definition of the TES site used?

Authors: Clarified in Materials and Methods, section "Gene selection" (lines 717-726).

Reviewer 1: Could the Authors clarify the TES site in their study, in the form of the size-window used (eg: Transcription End Site +/200bp). This specific information will greatly assist future readers in reproducing and interpreting the data.

added the missing information

Reviewer 1: 2. ATAC-seq changes occur at 8% of sites. The Authors note that more sites change in ATC-seq than in the RNA-seq - to highlight this, could the Authors indicate, in the relevant Results text, the % of total genes that are differentially-expressed with a similar threshold in young vs old by RNAseq?

Authors: Added in results, section "Aging has a modest effect on the transcriptional output" (lines 155-157).

Reviewer 1: The Authors have updated this very clearly.

Reviewer 1: 3. The data points are very small and/or the line colours are difficult to distinguish (Examples: Figures: 2C, 4A, S1E, S3A, S4C).

Authors: We have now made the lines thicker and hope that they are now more distinguishable.

Reviewer 1: The Authors have updated this very clearly.

Reviewer 1: 4. In Figure 3D the Authors note a cluster of genes that shows an average change in nascent transcription that appears decreased in middle-aged and old-aged mice (relative to young). However, the associated text describes that the cluster contains inflammatory genes, which might be expected to increase (Lines: 238-241) -could the Authors please clarify this part?

Authors: This is such a small cluster that made it impossible to perform GO enrichment (clarified this point now in the revised manuscript, lines 239-240). The few genes involved in interferon response also do not have a clear-cut phenotypes and there are reports in the literature, e.g. Nlrc5 has been reported to have anti-fibrogenic functions (PMID: 26806094). If the reviewer thinks that this part is too confusing, we would be happy to take out the reference to inflammation.

Reviewer 1: Cluster 4 is the smallest group in the analysis, reflecting the fewest genes, and therefore perhaps of the least importance. While this Cluster 4 contains some inflammatory genes, as the Authors explain, the GO term of inflammation is not enriched. So it is a minor point, and the Authors can choose to keep it in or remove the reference to it. I just want to advise that to me it seemed counter-intuitive that inflammatory genes would go down with aging, and it somewhat distracted from the main point of this panel.

We agree that this point might distract the reader from the main point of the figure. Therefore, we have removed any reference to inflammatory genes.

Reviewer 1: 5. Line 282-287: The Authors suggest that the Pausing Index (PI) is >1.5x in 69.7% of genes, and that similar levels of PI have been reported -can the Authors provide a reference for this? Several studies report a PI of >2x in ~90% of genes. If there is a difference in their study, can the Authors comment on that in the text?

Authors: We have added a reference in the results section "Promoter-proximal Pol II pausing decreased with age" (line 276). We have also re-analyzed using PI >2 as cut-off. In this case, 54.8 % of genes (1,453 out of 2,650) included in our analysis exhibit a PI >2. Due to the broad range of pausing indices among genes and the difficulty of applying discrete cut-offs to such a continuum, studies have reported estimates ranging from 30 to 90% of paused genes for multiple human and mouse cell types PMID: 22986266). This broad range is most likely a consequence of different experimental methods (ChIP-seq vs. nascent methods such as PRO- and NET-seq) and statistical criteria used to define Pol II pausing. Within this broad range, previous studies in HepG2 cells and mouse liver tissue found ~35% and ~50% of genes to exhibit a PI > 2 (PMID: 27259512), in line with our results. We chose a more fine-grained pausing classification, distinguishing between lowly, moderately and highly paused genes to investigate how these different pausing classes are affected by age. A study in bone marrow-derived macrophages using the same pausing classification found 76% (ChIP-seq) or 83% (PRO-seq) of genes to be highly or moderately paused, which is in a similar range as our results

Reviewer 1: This is very well explained, thank-you.

Reviewer 1: 6. Could the Author confirm the p-Values in some Figures. The value of 2,2 x 10-16 appears 6 times -is this correct? (Figures: 1D, 4B(twice), S2A, S7B(twice)

Authors: The programing language R uses this notation automatically for reporting very small numbers, as 2.2e-16 is the smallest number larger than 0 that can be stored by the floating system due to computer precision limits.

Reviewer 1: Thank-you, all is clear. I would only suggest to include this explanation somewhere in the Methods/Statistical section for the benefit of other readers.

As suggested by the reviewer, we have added this information to the “reproducibility and statistical analysis” part of the Method section.

Reviewer #2:

The authors did an outstanding job to address the reviewers' comments and improved the manuscript. I commend the authors for their careful and diligent work. I hope to see this published soon.

We thank the reviewer for these encouraging words and the support for publication.

Manuscript number: MSB-2022-11002RR
Title: Aging is associated with increased chromatin accessibility and reduced polymerase pausing in liver

Thank you again for sending us your revised manuscript. We are now satisfied with the modifications made and I am pleased to inform you that your paper has been accepted for publication.

EMBO Press Author Checklist

Corresponding Author Name: Peter Tessarz	
Journal Submitted to: Molecular Systems Biology	
Manuscript Number: MSB - 2022 - 11002	

USEFUL LINKS FOR COMPLETING THIS FORM
The EMBO Journal - Author Guidelines
EMBO Reports - Author Guidelines
Molecular Systems Biology - Author Guidelines
EMBO Molecular Medicine - Author Guidelines

Reporting Checklist for Life Science Articles (updated January
This checklist is adapted from Materials Design Analysis Reporting (MDAR) Checklist for Authors. MDAR establishes a minimum set of requirements in transparent reporting in the life sciences (see Statement of Task: 10.31222/osf.io/9sm4x). Please follow the journal's guidelines in preparing your
Please note that a copy of this checklist will be published alongside your article.

Abridged guidelines for figures
1. Data
The data shown in figures should satisfy the following conditions:
- the data were obtained and processed according to the field's best practice and are presented to reflect the results of the experiments in an accurate and unbiased manner.
- ideally, figure panels should include only measurements that are directly comparable to each other and obtained with the same assay.
- plots include clearly labeled error bars for independent experiments and sample sizes. Unless justified, error bars should not be shown for technical
- if n<5, the individual data points from each experiment should be plotted. Any statistical test employed should be justified.
- Source Data should be included to report the data underlying figures according to the guidelines set out in the authorship guidelines on Data

2. Captions
Each figure caption should contain the following information, for each panel where they are relevant:
- a specification of the experimental system investigated (eg cell line, species name).
- the assay(s) and method(s) used to carry out the reported observations and measurements.
- an explicit mention of the biological and chemical entity(ies) that are being measured.
- an explicit mention of the biological and chemical entity(ies) that are altered/varied/perturbed in a controlled manner.
- the exact sample size (n) for each experimental group/condition, given as a number, not a range;
- a description of the sample collection allowing the reader to understand whether the samples represent technical or biological replicates (including how many animals, litters, cultures, etc.).
- a statement of how many times the experiment shown was independently replicated in the laboratory.
- definitions of statistical methods and measures:
  - common tests, such as t-test (please specify whether paired vs. unpaired), simple χ2 tests, Wilcoxon and Mann-Whitney tests, can be unambiguously identified by name only, but more complex techniques should be described in the methods section;
  - are tests one-sided or two-sided?
  - are there adjustments for multiple comparisons?
  - exact statistical test results, e.g., P values = x but not P values < x;
  - definition of 'center values' as median or average;
  - definition of error bars as s.d. or s.e.m.

Please complete ALL of the questions below.
Select "Not Applicable" only when the requested information is not relevant for your study.

Materials

Newly Created Materials	Information included in the manuscript?	In which section is the information available? (Reagents and Tools Table, Materials and Methods, Figures, Data Availability Section)
New materials and reagents need to be available; do any restrictions apply?	Yes	Data Availability Section

Antibodies	Information included in the manuscript?	In which section is the information available? (Reagents and Tools Table, Materials and Methods, Figures, Data Availability Section)
For **antibodies** provide the following information:<br>- Commercial antibodies: RRID (if possible) or supplier name, catalogue number and or/clone number<br>- Non-commercial: RRID or citation	Yes	

DNA and RNA sequences	Information included in the manuscript?	In which section is the information available? (Reagents and Tools Table, Materials and Methods, Figures, Data Availability Section)
**Short novel DNA or RNA including primers, probes**: provide the sequences.	Not Applicable	

Cell materials	Information included in the manuscript?	In which section is the information available? (Reagents and Tools Table, Materials and Methods, Figures, Data Availability Section)
**Cell lines:** Provide species information, strain. Provide accession number in repository **OR** supplier name, catalog number, clone number, and/**OR** RRID.	Not Applicable	
**Primary cultures:** Provide species, strain, sex of origin, genetic modification status.	Yes	Materials and Methods
Report if the cell lines were recently **authenticated** (e.g., by STR profiling) and tested for mycoplasma contamination.	Not Applicable	

Experimental animals	Information included in the manuscript?	In which section is the information available? (Reagents and Tools Table, Materials and Methods, Figures, Data Availability Section)
**Laboratory animals or Model organisms:** Provide species, strain, sex, age, genetic modification status. Provide accession number in repository **OR** supplier name, catalog number, clone number, **OR** RRID.	Yes	Material and Methods
**Animal observed in or captured from the field:** Provide species, sex, and age where possible.	Not Applicable	
Please detail **housing and husbandry conditions**.	Yes	Material and Methods

Plants and microbes	Information included in the manuscript?	In which section is the information available? (Reagents and Tools Table, Materials and Methods, Figures, Data Availability Section)
**Plants:** provide species and strain, ecotype and cultivar where relevant, unique accession number if available, and source (including location for collected wild specimens).	Not Applicable	
**Microbes:** provide species and strain, unique accession number if available, and source.	Not Applicable	

Human research participants	Information included in the manuscript?	In which section is the information available? (Reagents and Tools Table, Materials and Methods, Figures, Data Availability Section)
If collected and within the bounds of privacy constraints report on age, sex and gender or ethnicity for all study participants.	Not Applicable	

Core facilities	Information included in the manuscript?	In which section is the information available? (Reagents and Tools Table, Materials and Methods, Figures, Data Availability Section)
If your work benefited from core facilities, was their service mentioned in the acknowledgments section?	Yes	Acknowledgements

Design

Study protocol	Information included in the manuscript?	In which section is the information available? (Reagents and Tools Table, Materials and Methods, Figures, Data Availability Section)
If study protocol has been **pre-registered, provide DOI in the manuscript**. For clinical trials, provide the trial registration number **OR** cite DOI.	Not Applicable	
Report the **clinical trial registration number** (at ClinicalTrials.gov or equivalent), where applicable.	Not Applicable	

Laboratory protocol	Information included in the manuscript?	In which section is the information available? (Reagents and Tools Table, Materials and Methods, Figures, Data Availability Section)
Provide DOI OR other citation details if **external detailed step-by-step protocols** are available.	Yes	References

Experimental study design and statistics	Information included in the manuscript?	In which section is the information available? (Reagents and Tools Table, Materials and Methods, Figures, Data Availability Section)
Include a statement about **sample size** estimate even if no statistical methods were used.	Not Applicable	
Were any steps taken to minimize the effects of subjective bias when allocating animals/samples to treatment (e.g. **randomization procedure**)? If yes, have they been described?	Not Applicable	
Include a statement about **blinding** even if no blinding was done.	Not Applicable	
Describe **inclusion/exclusion criteria** if samples or animals were excluded from the analysis. Were the criteria pre-established?<br><br>If sample or data points were omitted from analysis, report if this was due to attrition or intentional exclusion and provide justification.	Not Applicable	
For every figure, are **statistical tests** justified as appropriate? Do the data meet the assumptions of the tests (e.g., normal distribution)? Describe any methods used to assess it. Is there an estimate of variation within each group of data? Is the variance similar between the groups that are being statistically compared?	Yes	Figure legends

Sample definition and in-laboratory replication	Information included in the manuscript?	In which section is the information available? (Reagents and Tools Table, Materials and Methods, Figures, Data Availability Section)
In the figure legends: state number of times the experiment was **replicated** in laboratory.	Yes	
In the figure legends: define whether data describe **technical or biological replicates**.	Yes	

Ethics

Ethics	Information included in the manuscript?	In which section is the information available? (Reagents and Tools Table, Materials and Methods, Figures, Data Availability Section)
Studies involving **human participants**: State details of **authority granting ethics approval** (IRB or equivalent committee(s), provide reference number for approval.	Not Applicable	
Studies involving **human participants**: Include a statement confirming that **informed consent** was obtained from all subjects and that the experiments conformed to the principles set out in the WMA Declaration of Helsinki and the Department of Health and Human Services Belmont Report.	Not Applicable	
Studies involving **human participants:** For publication of **patient photos**, include a statement confirming that consent to publish was obtained.	Not Applicable	
Studies involving experimental **animals**: State details of **authority granting ethics approva**l (IRB or equivalent committee(s), provide reference number for approval. Include a statement of compliance with ethical regulations.	Yes	Materials and Methods
Studies involving **specimen and field samples:** State if relevant **permits** obtained, provide details of authority approving study; if none were required, explain why.	Not Applicable	

Dual Use Research of Concern (DURC)	Information included in the manuscript?	In which section is the information available? (Reagents and Tools Table, Materials and Methods, Figures, Data Availability Section)
Could your study fall under dual use research restrictions? Please check biosecurity documents and list of **select agents and toxins** (CDC): https://www.selectagents.gov/sat/list.htm	Not Applicable	
If you used a select agent, is the security level of the lab appropriate and reported in the manuscript?	Not Applicable	
If a study is subject to dual use research of concern regulations, is the name of the **authority granting approval and reference number** for the regulatory approval provided in the manuscript?	Not Applicable	

Reporting

The MDAR framework recommends adoption of discipline-specific guidelines, established and endorsed through community initiatives. Journals have their own policy about requiring specific guidelines and recommendations to complement MDAR.

Adherence to community standards	Information included in the manuscript?	In which section is the information available? (Reagents and Tools Table, Materials and Methods, Figures, Data Availability Section)
State if relevant guidelines or checklists (e.g., **ICMJE, MIBBI, ARRIVE, PRISMA**) have been followed or provided.	Not Applicable	
For **tumor marker prognostic studies,** we recommend that you follow the **REMARK** reporting guidelines (see link list at top right). See author guidelines, under 'Reporting Guidelines'. Please confirm you have followed these guidelines.	Not Applicable	
For **phase II and III randomized controlled trials**, please refer to the **CONSORT** flow diagram (see link list at top right) and submit the CONSORT checklist (see link list at top right) with your submission. See author guidelines, under 'Reporting Guidelines'. Please confirm you have submitted this list.	Not Applicable	

Data Availability

Data availability	Information included in the manuscript?	In which section is the information available? (Reagents and Tools Table, Materials and Methods, Figures, Data Availability Section)
Have **primary datasets** been deposited according to the journal's guidelines (see 'Data Deposition' section) and the respective accession numbers provided in the Data Availability Section?	Yes	Data Availability Section
Were **human clinical and genomic datasets** deposited in a public access-controlled repository in accordance to ethical obligations to the patients and to the applicable consent agreement?	Not Applicable	
Are **computational models** that are central and integral to a study available without restrictions in a machine-readable form? Were the relevant accession numbers or links provided?	Not Applicable	
If publicly available data were reused, provide the respective **data citations in the reference list**.	Yes	Data Availability Section, References
